# SHANK2 is a frequently amplified oncogene with evolutionarily conserved roles in regulating Hippo signaling

**DOI:** 10.1007/s13238-020-00742-6

**Published:** 2020-07-13

**Authors:** Liang Xu, Peixue Li, Xue Hao, Yi Lu, Mingxian Liu, Wenqian Song, Lin Shan, Jiao Yu, Hongyu Ding, Shishuang Chen, Ailing Yang, Yi Arial Zeng, Lei Zhang, Hai Jiang

**Affiliations:** 1 State Key Laboratory of Cell Biology, CAS Center for Excellence in Molecular Cell Science, Shanghai Institute of Biochemistry and Cell Biology, Chinese Academy of Sciences, University of Chinese Academy of Sciences, Shanghai, 200031 China; 2 School of Life Science and Technology, ShanghaiTech University, Shanghai, 201210 China; 3 Bio-Research Innovation Center, Shanghai Institute of Biochemistry and Cell Biology, Chinese Academy of Sciences, Suzhou, 215121 China

**Keywords:** SHANK2, oncogene, Hippo signaling, cancer

## Abstract

Dysfunction of the Hippo pathway enables cells to evade contact inhibition and provides advantages for cancerous overgrowth. However, for a significant portion of human cancer, how Hippo signaling is perturbed remains unknown. To answer this question, we performed a genome-wide screening for genes that affect the Hippo pathway in *Drosophila* and cross-referenced the hit genes with human cancer genome. In our screen, *Prosap* was identified as a novel regulator of the Hippo pathway that potently affects tissue growth. Interestingly, a mammalian homolog of *Prosap*, SHANK2, is the most frequently amplified gene on 11q13, a major tumor amplicon in human cancer. Gene amplification profile in this 11q13 amplicon clearly indicates selective pressure for SHANK2 amplification. More importantly, across the human cancer genome, SHANK2 is the most frequently amplified gene that is not located within the Myc amplicon. Further studies in multiple human cell lines confirmed that SHANK2 overexpression causes deregulation of Hippo signaling through competitive binding for a LATS1 activator, and as a potential oncogene, SHANK2 promotes cellular transformation and tumor formation *in vivo*. In cancer cell lines with deregulated Hippo pathway, depletion of SHANK2 restores Hippo signaling and ceases cellular proliferation. Taken together, these results suggest that SHANK2 is an evolutionarily conserved Hippo pathway regulator, commonly amplified in human cancer and potently promotes cancer. Our study for the first time illustrated oncogenic function of SHANK2, one of the most frequently amplified gene in human cancer. Furthermore, given that in normal adult tissues, SHANK2’s expression is largely restricted to the nervous system, SHANK2 may represent an interesting target for anticancer therapy.

**Electronic supplementary material:**

The online version of this article (10.1007/s13238-020-00742-6) contains supplementary material, which is available to authorized users.

## INTRODUCTION

In order to prevent malignant outgrowth, the number of cells in organs and tissues is tightly regulated. For normal cells in monolayer culture or in a tissue, proliferation is usually halted when cells reach high density (Gumbiner and Kim, 2014). As cell density increases, gradual changes in the cellular microenvironment, including cell-ECM and cell-cell interactions, cell shape and tension will impact cellular proliferation (Halder et al., 2012). Such a mechanism of “contact inhibition” is important for tissue homeostasis, and loss of contact inhibition is a hallmark of human cancer (Hanahan and Weinberg, 2011; Yu et al., 2015).

The Hippo signaling pathway is a primary responder for contact inhibition (Hanahan and Weinberg, 2011; Halder et al., 2012; Yu et al., 2015). First discovered in *Drosophila*, the Hippo pathway has been shown to significantly affect cell number and tissue growth (Harvey et al., 2003; Jia et al., 2003; Pantalacci et al., 2003; Udan et al., 2003; Wu et al., 2003; Huang et al., 2005). Later studies in mammalian system reached similar conclusions and showed that deregulation of the Hippo pathway causes cancer (Zender et al., 2006; Dong et al., 2007; Zhou et al., 2009; Atkins et al., 2016). The core components of Hippo pathway consist of upstream kinases MST1/2 and LATS1/2, which are activated by various upstream signals (Dupont et al., 2011; Wehr et al., 2013; Wang et al., 2015; Yang et al., 2015; Chakraborty et al., 2017) including cell-cell contact, and the downstream transcription cofactors YAP and TAZ, which promote cellular proliferation (Siew et al., 2008; Zhao et al., 2010; Halder and Johnson, 2011). Phosphorylation of YAP/TAZ by LATS1/2 leads to cytoplasmic sequestration and degradation of these transcription cofactors, thereby stopping cell growth and proliferation (Liu et al., 2010; Zhao et al., 2010).

Escaping from contact inhibition provides advantages for cancer cells, and facilitates tumorigenesis (Hanahan and Weinberg, 2011). However, analysis of human cancer genomes suggests that abnormalities of core components of the Hippo pathway, including mutations involving GNAQ (Yu et al., 2012; Feng et al., 2014), GNA11 (Yu et al., 2012, 2014) and NF2 (Xiao et al., 2003; Zhang et al., 2010; Yin et al., 2013), deletions involving VGLL4 (Jiao et al., 2014; Zhang et al., 2014), MST1 (Zhou et al., 2009; Song et al., 2010) and LATS1 (Yu et al., 2013), as well as amplifications involving YAP and TAZ (Overholtzer et al., 2006; Zender et al., 2006; Fernandez-L et al., 2009; Song et al., 2014), occur in a relatively small fraction of human cancer (Sanchez-Vega et al., 2018). Epigenetic silencing of MST1/2 and LATS1/2 has also been observed in mesothelioma (Maille et al., 2019) and sarcomas (Seidel et al., 2007; Merritt et al., 2018), as well as lung (Malik et al., 2018) and colorectal cancer (Wierzbicki et al., 2013). It remains unclear whether deregulation of other unknown components of the Hippo pathway may occur in human cancer and contribute to cancer growth.

In order to answer this question, we performed a genome-wide overexpression screen for novel Hippo pathway regulators in *Drosophila* and cross-referenced the screen hits with human cancer genome data to identify potential oncogenes with a role in Hippo signaling. Our results suggest SHANK2 is such an evolutionarily conserved regulator of Hippo pathway, commonly amplified in human cancer and potently promotes tumor formation.

## RESULTS

### Prosap overexpression causes tissue overgrowth via deregulation of Hippo signaling in Drosophila

We first searched for novel regulators of Hippo pathway in *Drosophila* using a screening system based on Hippo pathway’s regulation of tissue growth. In *Drosophila*, *yki* (ortholog of YAP) overexpression under the control of the *GMR-Gal4* driver (*GMR>yki*) causes an overgrown eye phenotype. This provides a sensitive platform for identifying additional Hippo pathway regulators (Huang et al., 2013; Hu et al., 2016) that may compound or ameliorate such an eye overgrowth phenotype.

**Figure 1:**
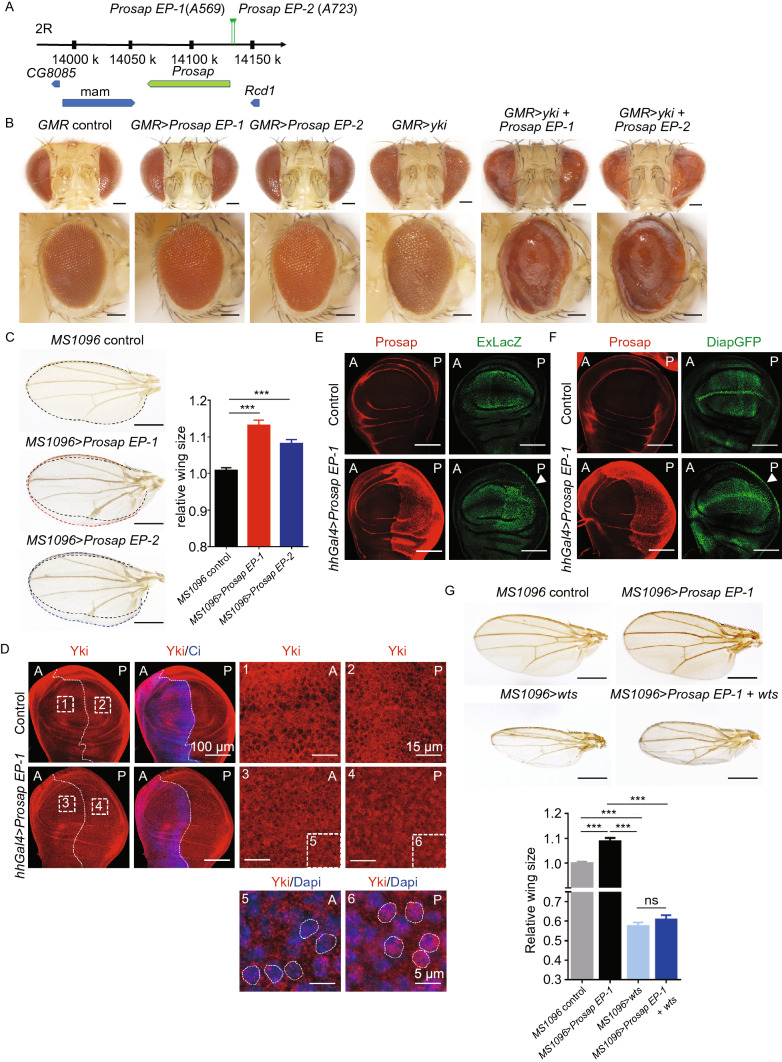
***Prosap*** **overexpression causes tissue overgrowth via deregulation of the Hippo pathway in** ***Drosophila.*** (A) The location of p-element insertion in *Prosap EP-1* (*A569*) and *Prosap EP-2* (*A723*) fly lines in chromosome. In both cases, insertions are located at 1,518 base pair upstream of the *Prosap* start codon. The inserted sequences in these two lines are different. (B) Eye overgrowth phenotype caused by *yki* expression was further enhanced by *Prosap EP-1* and *Prosap EP-2*. Shown are representative side views and dorsal views of eyes of indicated genotypes. Expression of *Prosap EP-1*, *Prosap EP-2* and *Yki* in *Drosophila* eyes was driven by *GMR-Gal4*. Scale bars: 100 μm. (C) Wing sizes increased in *MS1096* driven *Prosap EP-1* and *Prosap EP-2* lines. Shown are representative images of wings of indicated genotypes. Expression of *Prosap EP-1*, *Prosap EP-2* and *Yki* in *Drosophila* wings was driven by *MS1096-Gal4*. Scale bars: 500 μm. In the right panel, data represent mean ± SEM from results of three independent experiments; *n* = 13 for each group. *P* value was calculated by Student’s *t* test; ***P < 0.001. (D) Expression of *Prosap* caused nucleus localization of Yki. There are several isoforms encoded by the *Drosophila Prosap* gene. The *Prosap-PA* isoform was used in this study. Shown are representative images of *Drosophila* third-instar larval wing discs. *Prosap* overexpression was achieved in the posterior of wing discs in *hhgal4-Prosap EP-1 Drosophila*. Ci (blue) expressed in anterior wing discs was used to show the Anterior/Posterior boundary. The images on the bottom panel showed further magnification of Yki and DAPI staining. In posterior wing discs of *hhgal4-Prosap EP-1 Drosophila*, where *Prosap* was overexpressed, Yki mainly localized in the nucleus. White dotted lines were used to mark nucleus area stained by DAPI. A, anterior compartment; P, posterior compartment. (E and F) Overexpression of *Prosap* increased Yki transcriptional activity. Shown are representative images of *Drosophila* third-instar larval wing discs of indicated genotypes. The transcription level of Yki targets *expanded* and *diap1* were analyzed. In the fly strains used in this experiment, the expression of *LacZ* (E) or *GFP* (F) was driven by enhancers of *expanded* or *diap*, respectively. *Prosap* overexpression was achieved by hhGgal4 promoter in the posterior wing discs of *hhgal4-Prosap EP-1* flies. This led to moderately increased Yki activity in the center of posterior wing discs when compared to anterior wing discs. The edge of the posterior wing discs (arrowhead) showed significantly increased Yki activity. Scale bars: 100 μm. (G) *Wts* regulates tissue growth downstream of *Prosap.* Shown are representative images of wings of indicated genotypes. Expression of *Prosap EP-1* and *Wts* in *Drosophila* wings was driven by *MS1096-Gal4*. *Prosap* overexpression increased wing size, while co-expression of *Wts* suppressed the increase. Scale bars: 500 μm. In the lower panel, data represent mean ± SEM from results of three independent experiments; *n* = 9 for each group. *P* value was calculated by Student’s *t* test; ***P < 0.001

To perform the screen, we employed the p-element transposon system (Engels, 1996), which can induce gene overexpression when inserted into the promoter region (Rørth, 1996). We crossed 12,000 p-element inserted flies crossed with *GMR>yki* flies and searched for lines that could enhance the eye overgrowth phenotype induced by *yki* overexpression. Among the fly lines with the most pronounced effect in this screen, two independent *Drosophila* lines, A569 (EP-1) and A723 (EP-2) both exhibited p-element insertion at the 5′ UTR region of the *Prosap* gene (Fig. 1A). Both lines showed enhanced eye overgrowth phenotype induced by *yki* overexpression (Fig. 1B). These two lines also showed increased wing size, which is another phenotype associated with deregulated Hippo signaling activity (Fig. 1C) (Hu et al., 2016). Immunostaining experiments confirmed that in these two lines, *Prosap* was overexpressed (Fig. S1A and S1A’).

Interestingly, one of *Prosap*’s mammalian homologs, SHANK2, is highly amplified in human cancer. Therefore, we generated transgenic flies that overexpress *Prosap* to further confirm *Prosap*’s ability to promote tissue overgrowth. Consistent with the initial screen results, flies overexpressing *Prosap* caused a moderate eye overgrowth phenotype (Fig. S1B). *Prosap* overexpression also further enhanced the overgrowth phenotype caused by *GMR>yki* (Fig. S1B). In addition, we confirmed that in control flies the endogenous *Prosap* gene was expressed (Fig. S1C and S1D), and RNAi knockdown of *Prosap* caused reduction of wing size (Fig. S1E).

Next, we examined whether Prosap regulates Yki. Immunostaining of the imaginal wing discs of *Drosophila* third-instar larvae showed that *Prosap* overexpression caused Yki nuclear localization (Fig. 1D) and elevated transcriptional level of Yki transcriptional targets *expanded* and *Diap* (Fig. 1E and 1F), confirming that *Prosap* is a novel regulator of Hippo signaling.

Several additional lines of evidence suggest that *Prosap* functions in the Hippo pathway. First, overexpression of *wts* (orthology of LATS) suppressed *Prosap*’s ability to increase wing size (Fig. 1G). The wing size increase and eye overgrowth phenotypes of *Prosap*-overexpressing flies were also suppressed by *yki* RNAi (Fig. 1F and 1G). Lastly, *Prosap* knockdown could not suppress eye overgrowth phenotype induced by *yki* overexpression (Fig. S1B).

Taken together, these results established *Prosap* as a novel regulator of Hippo signaling in *Drosophila* and showed that its overexpression leads to tissue overgrowth.

### Overexpression of SHANK2 deregulates Hippo signaling activity in mammalian cells

In mammals, there are three *Prosap* homologs, SHANK1, SHANK2 and SHANK3 (Naisbitt et al., 1999; Hayashi et al., 2009). Of these three genes, SHANK2 is highly amplified in human cancer. According to TGCA copy number portal (Zack et al., 2013), 11% of human epithelial cancers exhibited focal amplification of SHANK2. In comparison, SHANK1 and SHANK3 are focally amplified each in 2% of human epithelial cancers (Table S1). Therefore we focused on the potential role of SHANK2 as a growth-promoting gene in human cancer.

**Figure 2:**
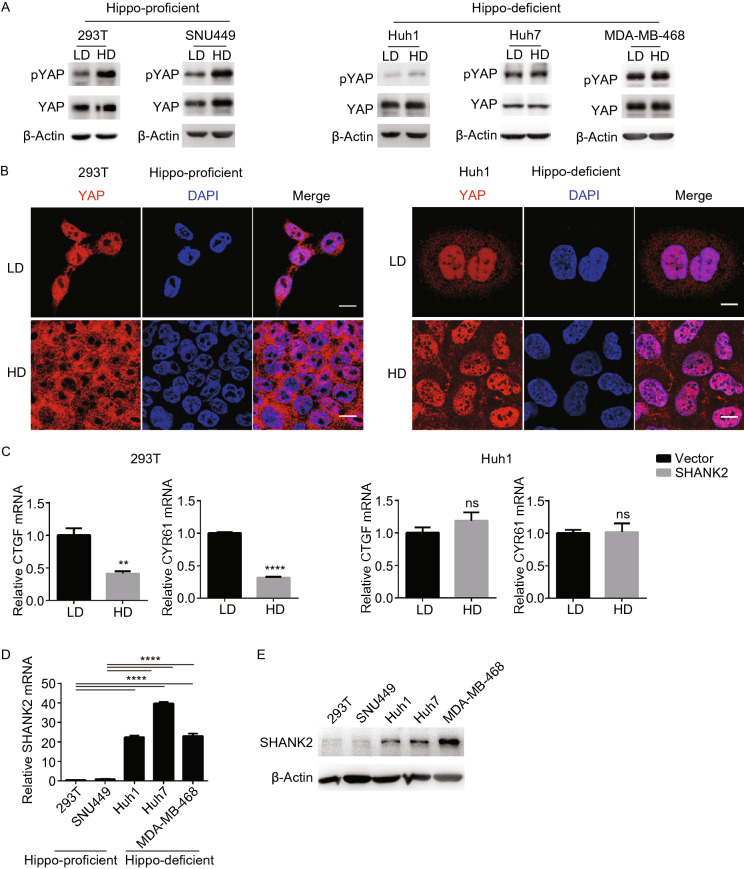
**Hippo signaling and SHANK2 expression in human cell lines.** (A and B) Analysis of human cell lines regarding their functional status of Hippo signaling. Hippo signaling was analyzed in human cell lines at low or high cell density (LD or HD) with indicated antibodies. Western blot analysis was used to analyze YAP phosphorylation status (A), and immunofluorescence was used to analyze subcellular localization of YAP. Scale bars: 10 μm (B). (C) Expression level of CTGF and CYR61 in 293T (Hippo-proficient) and Huh1 (Hippo-deficient) cell lines at low or high cell density (LD or HD). The expression level of CTGF and CYR61 was analyzed by qPCR. Data represent mean ± SEM from results of three independent experiments. P value was calculated by Student’s *t* test; **P < 0.01, ****P < 0.0001. (D and E) mRNA and protein level of SHANK2 in indicated human cell lines. For qPCR analysis in (D), data represent mean ± SEM from results of three independent experiments. *P* value was calculated by Student’s *t* test; ****P < 0.0001

First, we asked whether similar to its ortholog *Prosap* in *Drosophila*, SHANK2 also affects Hippo signaling in mammalian cells. We first analyzed human cell lines with regards to their Hippo pathway status and SHANK2 expression level. Cell lines are designated as Hippo-proficient if they are able to phosphorylate YAP and sequester YAP in cytoplasm at high cell density (Figs. 2A, 2B, and S2A). In contrast, Hippo-deficient cell lines fail to phosphorylate YAP, and YAP stays in nucleus at high cell density (Figs. 2A, 2B, and S2A). In addition, at high cell density, YAP transcription activities are low in Hippo-proficient cell lines and high in Hippo-deficient cell lines (Figs. 2C and S2B). Consistent with the finding that *Prosap* deregulates Hippo signaling in *Drosophila*, in two Hippo-proficient cell lines SHANK2 is not expressed, whereas in Hippo-deficient human cell lines SHANK2 is highly expressed (Fig. 2D and 2E).

**Figure 3:**
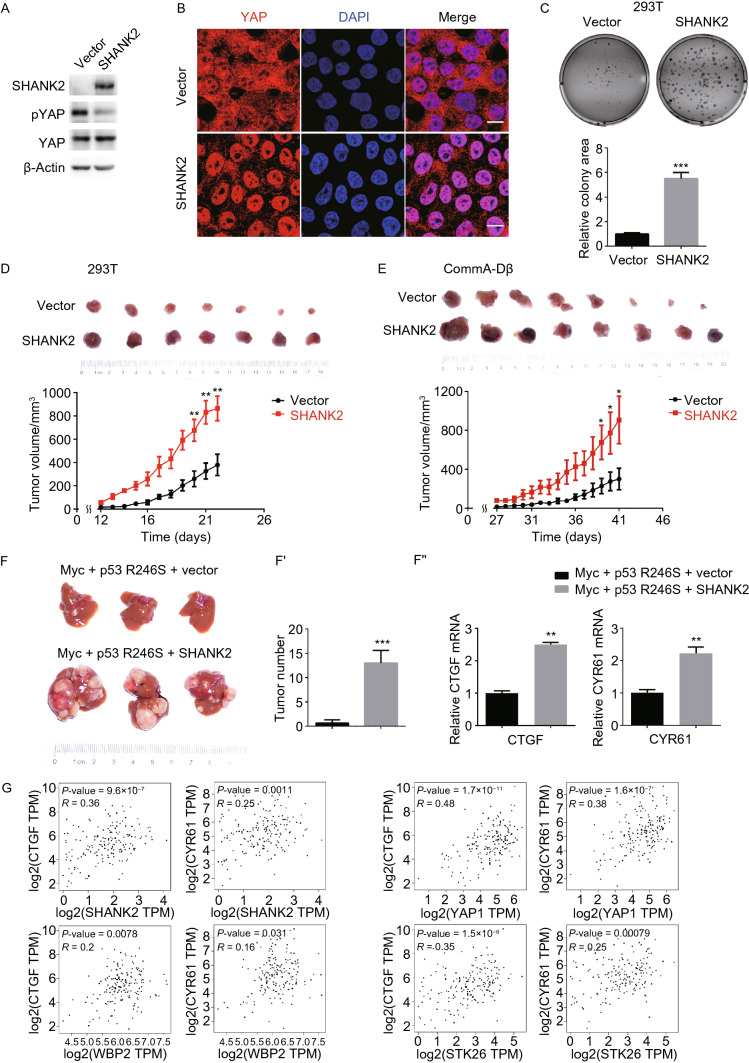
**SHANK2 deregulates Hippo pathway, transforms primary cells and promotes tumor growth.** (A) Ectopic expression of SHANK2 reduced YAP phosphorylation at high cell density in 293T cells. (B) In Hippo-proficient cell line 293T, ectopic expression of SHANK2 caused YAP to remain in nucleus at high cell density. Scale bars: 10 μm. (C) Expression of SHANK2 transformed 293T cells and enabled growth on soft agar. Lower panel, data represent mean ± SEM from results of three independent experiments. *P* value was calculated by Student’s *t* test; ***P < 0.001. (D and E) Expression of SHANK2 promoted tumor growth in nude mice. 2 million of 293T cells expressing vector control or exogenous SHANK2 were transplanted into nude mice (*n* = 7) (D). 2 million of CommA-Dβ mouse mammary cells expressing vector control or exogenous SHANK2 were transplanted into the fat pad of breast of nude mice (*n* = 8) (E). Measurement of tumor growth started after 12 days (D) or 27 days (E) post injection. Data present mean ± SEM. *P* value was calculated by Student’s *t* test; **P < 0.01, *P < 0.05. (F and F’’) Expression of SHANK2 promoted liver tumor formation in the context of Myc and p53 mutation. Liver tumors were induced in mice by hydrodynamically injecting the transposon vector control or exogenous SHANK2 combined with p53R246S and Myc. p53R246S is the murine version of p53R249S, a hotspot mutation commonly observed in human liver cancer. Shown are images of mice liver tumors (F), the statistics of tumor number (*n* = 3) (F’) and the expression level of YAP transcriptional target genes CTGF and CYR61 in tumors (F’’). Data represent mean ± SEM. *P* value was calculated by Student’s *t* test; **P < 0.001, ***P < 0.001. (G) Correlation of SHANK2 and CTGF, CYR61 expression in uterine corpus endometrial carcinoma (UCEC). The TCGA UCEC datasets were used for this analysis. Correlations between CTGF, CYR61 with YAP and two established positive regulators of YAP (WBP2 and STK26) were also shown in comparison

Next, we asked whether SHANK2 deregulates mammalian Hippo signaling and promotes cell growth. Ectopic expression of SHANK2 in Hippo-proficient cells suppressed YAP phosphorylation at high cell density (Fig. 3A). SHANK2 expression also caused YAP nuclear retention and high YAP activity despite high cell density (Figs. 3B, S3A, and S3B). This suggests that SHANK2 overexpression leads to deregulation of Hippo signaling in mammalian cells.

### SHANK2 potently promotes tumor growth

Next, we asked whether it functions as an oncogene to promote cancer. We first tested SHANK2’s ability to transform cells. In 293T cells, tumor suppressors Rb1 and p53 are inactivated by the SV40 large T antigen (Stepanenko and Dmitrenko, 2015), however, this cell line grows poorly in soft agar (Li et al., 2008). When SHANK2 was ectopically expressed in 293T cells, significant increased number of colonies formed in soft agar growth assay (Fig. 3C), demonstrating that SHANK2 indeed was able to transform human cells. SHANK2’s pro-growth effect likely depends on YAP activity, since YAP inhibition suppressed such phenotype (Fig. S3C). However due to the potential off-target effect of YAP inhibitor used in this experiment, we cannot rule out other possibilities. Lastly, when 293T cells were engrafted in nude mice, SHANK2 significantly enhanced growth *in vivo* (Figs. 3D and S4A).

In another experiment, we tested whether SHANK2 could promote *in vivo* growth of a mouse mammary cell line CommA-Dβ. Control or SHANK2-expressing cells were transplanted to mammary fat pad to analyze their rate of growth *in vivo*. The results showed that SHANK2 also significantly enhanced tumor growth in this model (Figs. 3E and S4B).

We further asked whether SHANK2 can promote tumor formation by endogenous cells in mice. Using a transposon system (Yant et al., 2004), murine versions of c-Myc and the p53 R246S dominant negative mutant were integrated into genomes of mouse liver cells via hydrodynamic injection. Such a genetic combination resulted in one small liver tumor in three mice. When SHANK2 is also included in the experiment, numerous huge liver tumors were observed in 4 weeks after hydrodynamic injection in all three mice, demonstrating that SHANK2 indeed potently promotes cancer formation *in vivo* (Fig. 3F and 3F’).

Importantly, in all three *in vivo* models, SHANK2-overexpressing tumors showed increased CTGF and CYR61 levels (Figs. 3F’’, S4C, and S4D), indicating enhanced YAP activity. In addition, immunostaining of the liver model showed that SHANK2 promotes YAP nuclear retention *in vivo* (Fig. S4E).

Lastly, we examined potential correlation between SHANK2 and CTGF,CYR61 expression levels. Analysis of uterine corpus endometrial carcinoma and esophageal carcinoma, two cancer types with the most prominent SHANK2 overexpression, showed that expression of SHANK2 positively correlates with CTGF and CYR61 (Fig. 3G). The degree of correlation is close to YAP-CTGF/CYR61 correlation. We also examined two established positive regulators of YAP, WBP2 (Lim et al., 2016) and STK26 (Sansores-Garcia et al., 2013) and found the degree of correlation between these two genes and CTGF/CYR61 is also close to SHANK2-CTGF/CYR61 correlation (Fig. S4F). Such correlations provide further support to our hypothesis that SHANK2 positively regulates YAP in cancer. Taken together, our results strongly support SHANK2’s role as a novel oncogene that deregulates Hippo pathway.

### SHANK2 interferes with Hippo signaling through sequestration of ARHGEF7

Next, we investigated the mechanism by which SHANK2 deregulates Hippo signaling. It is known that YAP is phosphorylated and inactivated by LATS1/2. SHANK2 is an actin-associated scaffold protein primarily expressed in nervous system (Naisbitt et al., 1999). Previous study reported that SHANK2 interacts with β-PIX/ARHGEF7 at synapses in cultured neurons (Park et al., 2003). Interestingly, it was recently shown that ARHGEF7 interacts with LATS1 and YAP, and functions as an platform for LATS1-mediated YAP phosphorylation (Heidary Arash et al., 2014). Based on these two studies, we hypothesized that in cancer cells, overexpressed SHANK2 interacts with and sequesters ARHGRF7 away from LATS1, which then leads to reduced LATS1 activity and enhanced cell growth.

**Figure 4:**
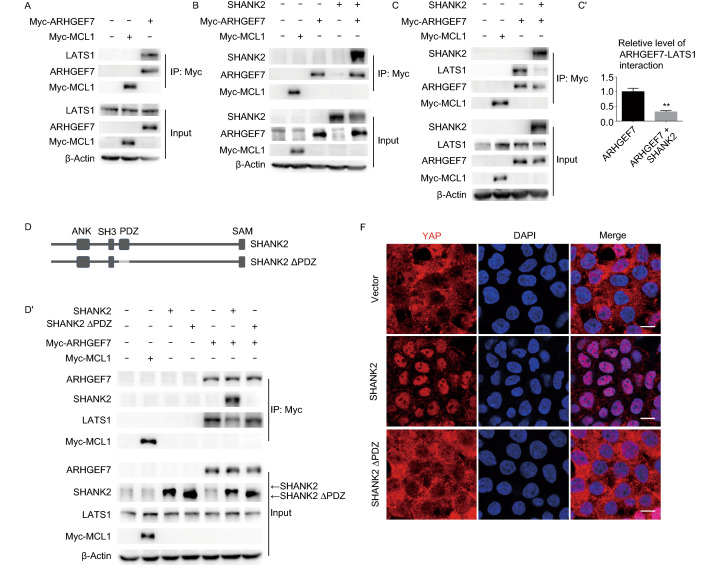

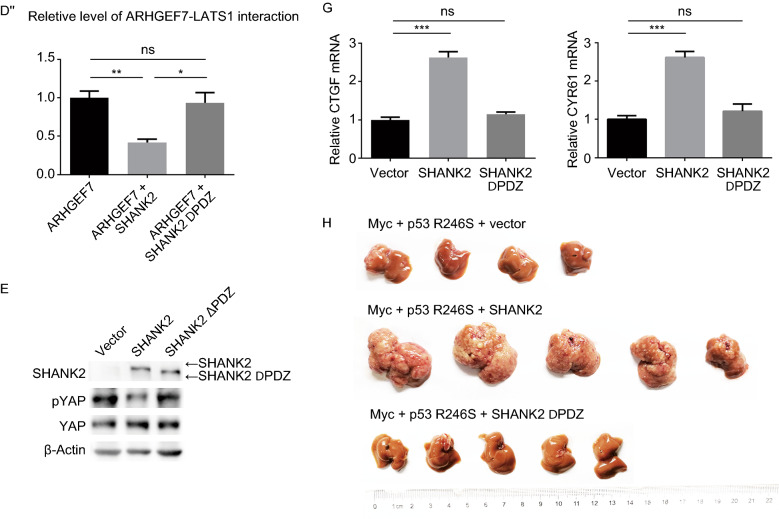
**SHANK2 deregulates Hippo pathway by antagonizing LATS1 activity through sequestration of ARHGEF7.** (A) Co-immunoprecipitation of ARHGEF7 and LATS1. (B) Co-immunoprecipitation of ARHGEF7 and SHANK2. (C and C’) SHANK2 overexpression reduced ARHGEF7-LATS1 interaction. For data shown in (A) to (C), Myc-MCL1 was used as negative control and the experiments were done in 293T cells. The relative amount of ARHGEF7-LATS1 interactions were quantitated in (C’) as mean ± SEM from results of three independent experiments. *P* value was calculated by Student’s *t* test; **P < 0.01. (D and D’’) The PDZ domain of SHANK2 mediated SHANK2-ARHGEF7 interaction and was crucial for SHANK2’s ability to disrupt ARHGEF-LATS1 interaction. A schematic representation of SHANK protein domains was shown in (D). Deletion of PDZ domain (SHANK2 ΔPDZ) rendered SHANK2 unable to bind ARHGEF7 and abolished SHANK2’s ability to disrupt ARGHEF7-LATS1 interaction (D’). The relative amount of ARHGEF7-LATS1 interactions were quantitated in (D’’) as mean ± SEM from results of three independent experiments. *P* value was calculated by Student’s *t* test; *P < 0.05, **P < 0.01. (E and F) Deletion of PDZ domain rendered SHANK2 unable to reduce YAP phosphorylation (E) or promote YAP nuclear localization (F) in high cell density. Experiments were done in 293T cells. Scale bars: 10 μm. (G) CTGF and CYR61 expression level in control, SHANK2 and SHANK2 ΔPDZ groups. Experiments were done in 293T cells. Data represent mean ± SEM from results of three independent experiments. *P* value was calculated by Student’s *t* test; ***P < 0.001. (H) Deletion of PDZ domain abolished SHANK2’s ability to promote liver cancer formation *in vivo*. Vector control or SHANK2, ΔPDZ SHANK2 combined with p53R246S and Myc were hydrodynamically injecting into mouse tail vain to induce liver cancer

Through Co-immunoprecipitation assays, we confirmed that both LATS1 and SHANK2 interact with ARHGEF7 (Fig. 4A and 4B). Importantly, upon SHANK2 overexpression, significantly less amount of ARHGEF7 interacts with LATS1 (Fig. 4C and 4C’) and YAP (Fig. S5). Therefore, overexpressed SHANK2 is indeed capable of sequestering ARHGEF7 from LATS1 and YAP.

Of note, the *Drosophila* ortholog gene of ARHGEF7, *Pix*, has been shown to activate *Hpo* kinase, the homologous of MST (Dent et al., 2015). This, and the ARHGEF7-LATS interaction in mammalian cells (Heidary Arash et al., 2014) (Fig. 4A) suggest that ARHGEF7/*Pix* functionally interacts with the Hippo pathway core kinases, but the mechanism of regulation may slightly diverge between species.

Consistent with previous report (Park et al., 2003), we found the PDZ domain of SHANK2 is crucial for its interaction with ARFGEF7 (Fig. 4D, 4D’, and 4D’’). Importantly, ΔPDZ SHANK2 could not interfere with ARHGEF7-LATS1 binding (Fig. 4D’ and 4D’’). Deletion of PDZ from SHANK2 also diminished its ability to deregulate the phosphorylation, localization and activity of YAP (Fig. 4E–G) and to promote liver cancer formation *in vivo* (Fig. 4H). Based on these experimental results, we speculate that overexpressed SHANK2 caused sequestration of ARHGEF7, resulting in decreased YAP phosphorylation and deregulated Hippo signaling.

### Cancer cell lines that overexpress SHANK2 are dependent on SHANK2 for growth

**Figure 5:**
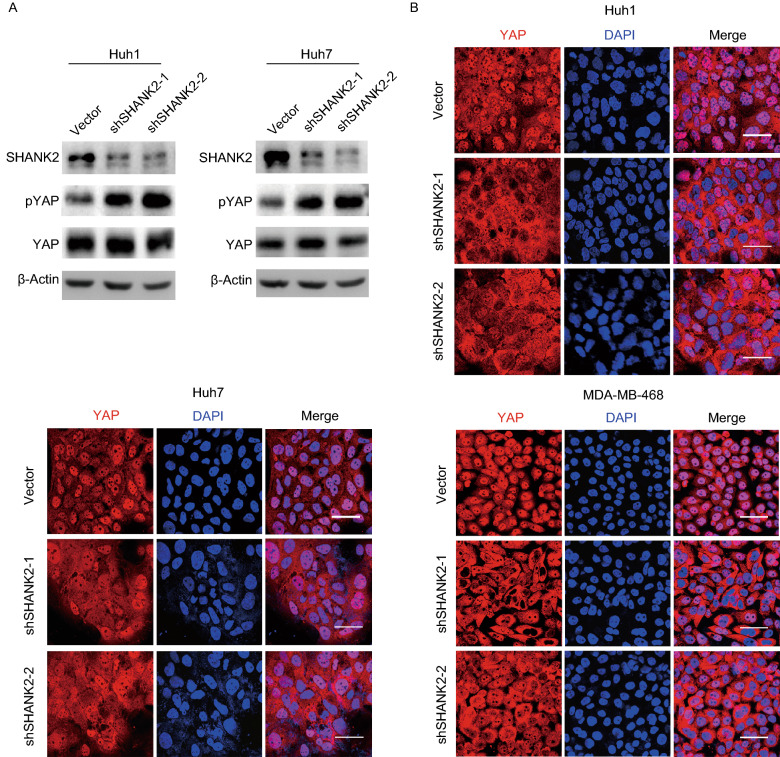
**Restoration of Hippo signaling upon depletion of SHANK2.** (A) In Hippo-deficient cancer cell lines Huh1 and Huh7, shRNA-mediated knockdown of SHANK2 restored YAP phosphorylation at high cell density. (B) Knockdown of SHANK2 caused cytoplasm retention of YAP at high cell density in Hippo-deficient cancer cell lines. Scale bars: 35 μm

**Figure 6:**
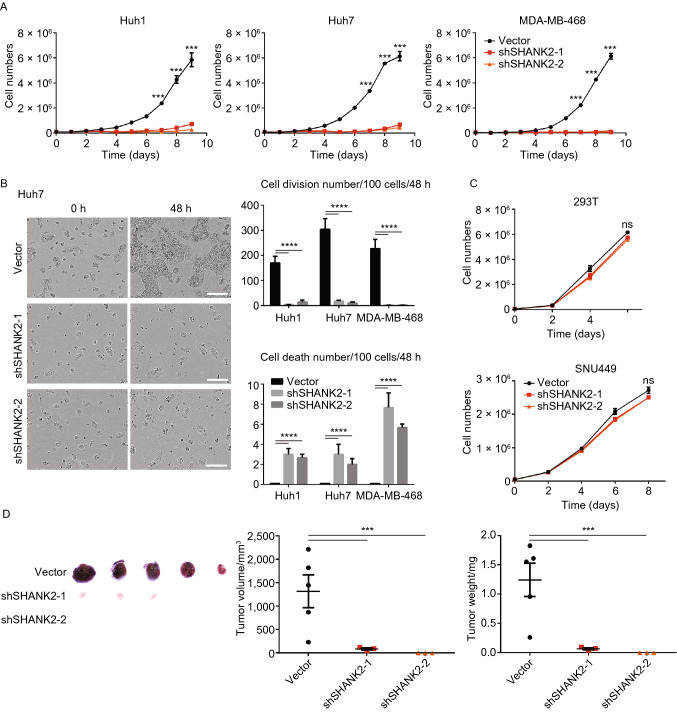
**SHANK2 knockdown inhibits growth of SHANK2 high-expressing cell lines.** (A) SHANK2 shRNA resulted in reduced cell number of Huh1, Huh7 and MDA-MB-468, three cancer cell lines that showed high expression of SHANK2 and defective Hippo signaling. Cells were plated at 0.5 × 10^5^cells/plate and counted each day. Data represent mean ± SEM from results of three independent experiments. *P* value was calculated by Student’s *t* test; ***P < 0.001. (B) The impact of SHANK2 knockdown on cell proliferation and cell death. Real-time imaging was performed for Huh1, Huh7 and MDA-MB-468 that express control vector or SHANK2 shRNA. From each video, three different areas containing ~100 cells were counted for cell division and cell death events during 48 h. Scale bars: 200 μm. In the left panel, data represent mean ± SEM. *P* value was calculated by Student’s *t* test; ****P < 0.0001. (C) SHANK2 shRNA had minimal effects on cellular proliferation in 293T and SNU449, which express low levels of SHANK2. Cells were plated at 0.5 × 10^5^cells/plate and counted every other day. Data represent mean ± SEM from results of three independent experiments. *P* value was calculated by Student’s *t* test. ns, not significant. (D) SHANK2 knockdown suppressed tumor growth *in vivo.* 2 million of Huh7 cells expressing vector control or SHANK2 shRNA were transplanted into nude mice, and tumor volume and weight were analyzed at day 27 post injection. Data represent mean ± SEM; *n* = 5. *P* value was calculated by Student’s *t* test; ***P < 0.001

Lastly, we examined cancer cell lines that overexpress SHANK2 and exhibit deregulated Hippo signaling. We asked whether knockdown of SHANK2 could restore Hippo signaling in these cell lines and reduce their proliferation. In multiple human cancer cell lines that overexpress SHANK2, upon SHANK2 knockdown, YAP phosphorylation was restored at high cell density, and YAP were sequestered in cytoplasm under such conditions (Fig. 5A and 5B). This indicated that SHANK2 knockdown restored Hippo signaling in such cells. Moreover, knockdown of SHANK2 resulted in significantly reduced cell number in these cell lines (Fig. 6A). Live cell imaging of these cells indicated that upon SHANK2 knockdown, cellular proliferation was significantly suppressed (Fig. 6B). A small number of cells also underwent cell death over time (Fig. 6B). In contrast, SHANK2 shRNA had little effect on the proliferation of Hippo-proficient cell lines (Fig. 6C). When injected into nude mice, SHANK2 depletion also severely reduced the ability to form tumor *in vivo* (Fig. 6D). In many cases, after SHANK2 depletion no cancer cell mass was discovered *in vivo*. These data further supported SHANK2’s role in Hippo signaling, and suggested SHANK2 may provide a potential target for treating cancer.

### SHANK2 is prominently amplified in human cancer

**Figure 7:**
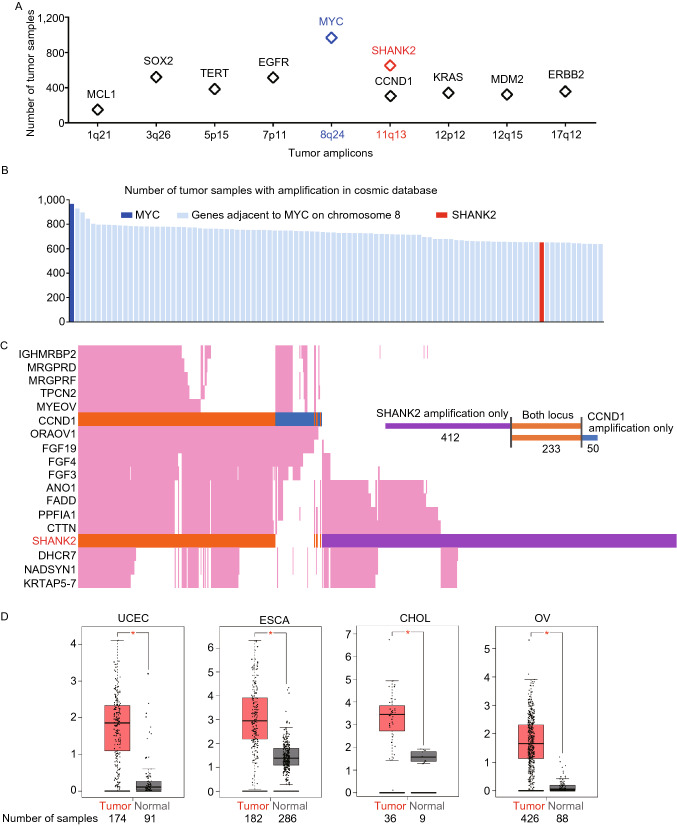
**SHANK2 is highly amplified and overexpressed in human cancer.** (A) SHANK2 on 11q13 is one of the most frequently amplified genes in human cancer. Shown are the numbers of tumor samples with amplification of major oncogenes. Data were tallied from COSMIC database. (B) The top 100 most frequently amplified genes in human cancer. Gene amplification status of all human genes were tallied from the COSMIC database and ranked with amplification frequency. Except for SHANK2, all other genes on the top 100 list are on the Myc amplicon at Chromosome 8. (C) Amplification status of 11q13 genes in tumor samples according to COSMIC database. On the Y axis, genes in 11q13 are aligned according to their chromosomal location. On the X axis, each line represents one tumor sample. Tumor samples that amplify both SHANK2 and CCND1 (cyclin D1) are shown in orange. Tumor samples that amplify SHANK2 but not CCND1 are shown in purple. Tumor samples that amplify CCND1 but not SHANK2 are shown in blue. The results indicate there are separate selective pressures for SHANK2 and CCND1 amplification in human cancer. (D) Multiple types of human cancer overexpress SHANK2. Analysis of tumor (red) vs. normal tissue (black) was done base on TCGA dataset. UCEC: uterine corpus endometrial cancer; ESCA: esophageal cancer; CHOL: cholangiocarcinoma; OV: ovarian cancer. *P < 0.05.

To further understand the relative significance of SHANK2 amplification in human cancer, we referenced the COSMIC (Catalogue Of Somatic Mutations In Cancer) database, which provided gene amplification information of approximately 15,000 cancer samples. Interestingly, judging by the number of cancer samples carrying gene amplification, SHANK2 was more frequently amplified than many well-established oncogenes (Fig. 7A).

To more clearly estimate the significance of SHANK2 amplification in human cancer, we compiled gene amplification status for all human coding genes based on the COSMIC dataset. In human cancer, Myc is the most frequently amplified gene. Many genes that are neighboring Myc on chromosome 8q are also significantly co-amplified in cancers. Strikingly, out of the 100 most frequently amplified genes in human cancer, SHANK2 is the only exception that does not reside on chromosome 8q (Fig. 7B, and Table S2). Such a striking amplification status for SHANK2 suggests that it’s a very prominent genomic event for human cancer. Since SHANK2’s ortholog *Prosap* promotes tissue overgrowth in *Drosophila*, such genomic data suggest that SHANK2 may functions as an important oncogene. To our knowledge, so far no studies have shown SHANK2 promotes cancer formation.

SHANK2 is located at the 11q13 tumor amplicon, a relatively large amplicon containing several focal amplification peaks. Cyclin D1, which drives cell cycle progression, is also located in one of such amplification peaks. The TCGA copy number portal database showed Cyclin D1 is located in an amplification peak that only contains Cylcin D1 and ORAOV1. This suggests that during cancer formation, SHANK2 is selected independent of Cyclin D1.

To further understand this, we performed a detailed analysis of the SHANK2 and Cyclin D1 amplification status in COSMIC tumor samples. There appears to be separate amplification peaks involving SHANK2 and Cyclin D1 (Fig. 7C). Among the 11q13-amplified cancer samples that carry amplification of SHANK2 and/or Cyclin D1, 233 amplified both SHANK2 and Cyclin D1, 412 amplified only SHANK2 and 50 amplified only Cyclin D1 (Fig. 7C and Table S3). This suggests that there are separate selective pressures for SHANK2 and Cyclin D1 amplification, and they may both promote cancer. Importantly, in addition to amplification, SHANK2 is also overexpressed in multiple types of human cancer (Fig. 7D) (Tang et al., 2017). A recent study of esophageal squamous cell carcinoma from South Africa also confirmed SHANK2 overexpression via immunohistochemistry staining (Brown et al., 2020). In their analysis, focal amplification of 11q13.3 was observed in 37% of cancer samples and 79% of these samples showed overexpression of SHANK2. Together with our experimental data, this further strengthens a role for SHANK2 in promoting cancer. Given that in human cancer, SHANK2 is the most frequently amplified gene outside of the Myc amplicon, it may provide explanation for how a significant portion of human cancer disables Hippo signaling and evades contact inhibition.

## DISCUSSION

In this report, we present multiple lines of evidence supporting SHANK2’s potential role as a novel oncogene that affects Hippo signaling. Genetically, overexpression of SHANK2’s ortholog *Prosap* deregulates Hippo signaling and promotes tissue overgrowth in *Drosophila*. Genomically, SHANK2 is the most frequently amplified gene outside the Myc amplicon in human cancer. Both our analysis of the COSMIC dataset (Fig. 1C) and the Broad cancer gene copy number analysis (Table S1) of the 11q13 tumor amplicon clearly indicates a selection for SHANK2 amplification. Biochemically, SHANK2 regulates Hippo signaling, and its overexpression leads to YAP activation and cellular transformation. Taken together, these results indicate SHANK2 is an evolutionarily conserved regulator of Hippo signaling with oncogenic function in human cancer.

In the Hippo pathway, SHANK2 functions as an upstream regulator. In both *Drosophila* and human cells, depletion or inhibition of YAP/*yki* blocked the pro-growth effect of SHANK2 (Fig. S1F, S1G, and S3C). Given that many cancers amplify SHANK2, this finding may help understand how cancer cells managed to escape from contact inhibition. Our results, and the recent finding that cancerous SWI/SNF mutations cause YAP activation (Chang et al., 2018) will further expand the picture of Hippo pathway’s broad involvement in human cancer.

Prior to our study, there were no reports demonstrating SHANK2’s oncogenic role in human cancer. Several studies noted SHANK2’s amplification in esophageal and oropharyngeal cancer and its association with poor prognosis (Carneiro et al., 2008; Qin et al., 2016; Barros-Filho et al., 2018; Yu et al., 2019; Brown et al., 2020). For example, in a recent analysis of esophageal cancer, a tumor type with frequent 11q13 amplification, SHANK2 overexpression was identified as one of the most significant factors for poor patient survival, second only to tumor stage (Qin et al., 2016). These findings further suggest that SHANK2 plays an important role in cancer.

**Figure 8:**
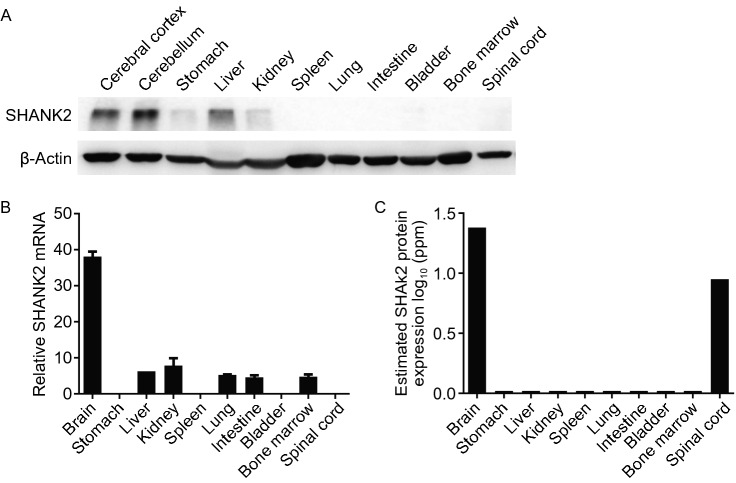
**SHANK2 expression is mostly restricted to the neuronal system.** (A) SHANK2 expression in adult mice tissues determined by Western blot. (B) mRNA expression of SHANK2 in normal human tissues from BioGPS. Data was obtained for 76 normal human tissues and compartments hybridized against GeneAtlas U133A. (C) Protein expression of SHANK2 in normal human tissues from HIPED (the Human Integrated Protein Expression Database)

As a novel oncogene, SHANK2 could potentially provide a new target for treating cancer. SHANK2 mutation has been recently linked to autism (Berkel et al., 2010; Won et al., 2012; Schneider et al., 2014), and SHANK2 expression is mostly restricted to the nervous system (Naisbitt et al., 1999; Hayashi et al., 2009; Berkel et al., 2010; Won et al., 2012; Schneider et al., 2014). Immunoblot analysis of various mouse tissues confirmed the lack of expression of SHANK2 in most non-neuron tissues, including bone marrow and intestine, two major sites of toxicities for cancer treatment (Fig. 8A). Querying of public databases (Pontén et al., 2011; Wu et al., 2016) suggests the expression pattern of SHANK2 in human is similar to mouse, and protein expression of SHANK2 is mainly observed in brain and spinal cord (Fig. 8B and 8C).

Consistent with such expression pattern, full-body SHANK2 knockout mice showed expected neuronal phenotypes and smaller body size but were otherwise normal (Schmeisser et al., 2012; Won et al., 2012). Although low level of SHANK2 expression was observed in mouse liver (Fig. 8), no liver-associated phenotypes were reported for the SHANK2 knockout mice, suggesting that SHANK2 may not be needed for liver function under physiological conditions. Whether SHANK2 is needed in liver under pathological conditions (regeneration after resection and liver damage) remains to be tested in such mice.

Considering the expression pattern of SHANK2 and the knockout mice phenotype, it is possible that SHANK2 is necessary for growth only in cancer cells that overexpress SHANK2, and that most human normal adult tissues do not utilize SHANK2 for growth. Therefore, therapies targeting SHANK2 could potentially be achieved with low toxicity. Given that SHANK2 is a scaffold protein (Naisbitt et al., 1999; Hayashi et al., 2009; Berkel et al., 2010; Won et al., 2012; Schneider et al., 2014), chemically inhibiting its function may prove difficult. However, with the recent advances in siRNA and antisense oligos therapies (Stein and Castanotto, 2017; Setten et al., 2019), it is possible to suppress SHANK2 with these approaches. Vehicles that spare the nervous system could be utilized to limit the side effects of such SHANK2-targeting siRNA and antisense oligos. Alternatively, targeted protein degradation techniques (Gadd et al., 2017) can be utilized. It may be possible to generate PROTACs (proteolysis targeting chimeric molecules) that target SHANK2 for degradation, but do not pass the blood brain barrier. Such drugs could be useful in treating cancers that depend on SHANK2. Given the recent finding that 11q13 amplification does not sensitize cancers to CDK4/6 inhibitor (Li et al., 2018), SHANK2 may present an alternative target for treating cancers with 11q13 amplification.

Among the three mammalian homologs of *Prosap*, SHANK2 is the most prominently amplified gene in human cancer. About five-fold more cancer samples exhibit SHANK2 amplification compared to SHANK1 and SHANK3. The protein domains of these three SHANK genes are very similar (Fig. S6), however due to the big size of these proteins and the difficulty to clone them, we only focused on SHANK2 in this study. We cannot speculate whether SHANK1 and SHANK3 similarly interfere with Hippo signaling. If they do, they may also represent potentially interesting therapy targets, since their expression pattern and knockout mice phenotypes (Hung et al., 2008; Peça et al., 2011; Wang et al., 2011) are similar to those of SHANK2.

With regard to how SHANK2 regulates Hippo signaling, our study points to a possible explanation that SHANK2 disrupts the interaction between LATS1 and ARHGEF7. Interestingly, SHANK2 is an actin cytoskeleton bundling protein, and it is known that actin affects Hippo signaling (Dupont et al., 2011; Yu et al., 2012; Zhao et al., 2012). Therefore, other possibilities as to how SHANK2 affect Hippo pathway also exists. For example, considering SHANK2’s interaction with the actin cytoskeleton, its overexpression may also affect cell junctions to alter Hippo signaling. Our data suggests that deletion of PDZ domains from SHANK2 abolished its ability to regulate LATS1/2. It is possible that aside from ARHGEF7,other cell junction proteins may also interact with the PDZ domain of SHANK2 and contribute to such phenotype. This remains a question for further research.

Of note, the molecular events from actin to Hippo signaling remain a subject of study, our study of the SHANK2-ARHGEF7-LATS1 interactions provided such a possible route. Considering that in human SHANK2 is not expressed in most adult tissues, such a mechanism may be more relevant to pathological conditions, when SHANK2 is amplified in cancers. On the other hand, knocking down of SHANK2 in several cell lines led to severe block of cell growth (Fig. 6A). It is possible that mechanisms other than LATS1/2 inhibition also contributed to such a dramatic phenotype.

Taken together, our study for the first time assigned an oncogenic function for SHANK2, one of the most prominently amplified genes in human cancer. Our results indicate that SHANK2 is an evolutionarily conserved regulator of Hippo signaling. This study provides further insight into how cancer cells deregulate Hippo signaling and evade contact inhibition, and points to a potential intervention target for cancer therapy.

## Electronic supplementary material

Below is the link to the electronic supplementary material.

Supplementary material 1 (PDF 1276 kb)
