# Supplementary material for: SHANK2 is a frequently amplified oncogene with evolutionarily conserved roles in regulating Hippo signaling
Source: Protein Cell. 2020 Jul 13;12(3):174–93. doi: 10.1007/s13238-020-00742-6 (PMC7895894; doi:10.1007/s13238-020-00742-6)
Supplement: Supplementary file 1 — Supplementary material 1 (PDF 1276 kb) [file 13238_2020_742_MOESM1_ESM.pdf]

Supplementary materials

Materials and Methods

p-element insertion screen, Cloning, Transgenes, *Drosophila* stocks, Immunostaining and Microscopy in *Drosophila*

p-element insertion screen

We performed a gain-of-function EP lines screen (Rørth, 1996). The EP library for the screen was a gift from Jianming Chen (Third Institute of Oceanography, State Oceanic Administration, China). Each EP line was crossed with GMR-Yki flies, and we searched for lines that could enhance the eye overgrowth phenotype induced by yki overexpression. From more than 10,000 EP lines (Huang et al., 2013),we identified several lines with pronounced effect and analyzed the UAS element insertion sites of these lines. Two independent lines, A569 and A723, both exhibited P-element insertion at the 5'UTR region of the Prosap gene.

Cloning, Transgenes, *Drosophila* stocks and Immunohistochemistry

Prosap cDNA was amplified with PCR from BDGP DGC clones (LD13733) and introduced into *pUAST-Myc* vector. Transgenic flies expressing *Prosap* was generated by microinjection.

The following transgenes were used in this study: *Prosap* RNAi (VDRC21218) and *yki* RNAi (VDRC40497). Other stocks used in this study, including *ExlacZ, DiapGFP, hhGal4, GMR-Gal4, MS1096-Gal4,* and *UAS-yki*, have all been previously described (Zhang et al., 2008). All the flies were cultured at 25℃.

Immunostaining experiments were done on *Drosophila* third-instar larval wing discs or *Drosophila* eyes. The procedures for immunostaining of imaginal discs were previously described (Zhang et al., 2008).The antibodies used in this study include rabbit anti-β-gal (1: 500, Invitrogen), Rat anti-cubitus interruptus (Ci) (Developmental Studies Hybridoma Bank, DSHB), rabbit anti-Yki (1:50, produced by immunizing

rabbits with the Yki peptide of amino acids 180-418) (Guo et al., 2013) and Rabbit anti-Prosap (1:100, produced in Dr. Lei Zhang's lab by immunizing rabbits with the peptide of Prosap amino acids 701-1300).

Microscopy

Fluorescent microscopy was performed on a Leica LAS SP8 confocal microscope; confocal images were obtained using the Leica AF Lite system under 40× objective. For immunofluorescence staining, single layer image is shown.

Analysis of gene amplification status in cancer database

We gathered information on copy number amplification for all human genes from the COSMIC website. Gene copy number results from about 15,000 cancer samples are available from COSMIC, which forms the basis of our analysis. To systematically analyze such data, we used the following method. First, cancer type-specific summary of gene amplification dataset was downloaded from COSMIC. For example, on the following webpage https://cancer.sanger.ac.uk/cosmic/browse/tissue?wgs=off&sn=breast&ss=&hn=&sh=&in=t&src=tissue&all_data=n at the "variant" section, information for all genes with amplification in breast cancer can be found. Out of 1544 breast cancer samples, 234 exhibited amplification for TRPS1, 232 for EIF3H and so on. We downloaded such data from all cancer types listed on COSMIC using the above method. Next, for each individual gene, the number of cancer samples carrying its amplification was tallied from each cancer type. The top 100 most frequently amplified genes in human cancer are listed in Supplemental Table 2, and SHANK2 is the most frequently amplified gene outside the Myc amplicon. Data used in our analysis in Figure 2 were downloaded from COMSIC database in June 2017.

For Supplemental Table 3, the list of cancer samples exhibiting high value amplification for CCND1 and SHANK2 can be accessed at http://cancer.sanger.ac.uk/cosmic/cnv/details?all_data=&chr=11&cnv=gain&coords=

AA%3AAA&dr=&end=69643991&gd=&id=3241&ln=CCND1&seqlen=296&start=69641314

and

http://cancer.sanger.ac.uk/cosmic/cnv/details?all_data=&chr=11&cnv=gain&coords=AA%3AAA&dr=&end=70487720&gd=&id=61288&ln=SHANK2&seqlen=1255&start=7049027

In the above links, a small number of cancer samples were listed more than once. We manually removed such redundancies before generating Supplemental Table 3. Due to COSMIC updates, the current number on the above pages are slightly higher than the numbers in our analysis, which was done based on COSMIC database as of June 2017.

Additional analysis of gene amplification status (Supplemental Table 1) was performed with the TCGA Copy Number Portal website. http://portals.broadinstitute.org/tcga/home

Cell lines and tissue culture

293T, Huh1, Huh7, SNU449 , MDA-MB-468 and CommA-Dβ cells were cultured in DMEM (Hyclone) containing 10% FBS (Gibco) at 37℃ with 5% CO_2 .

Reagents and Antibodies

Antibodies against SHANK2 (Santa Cruz Biotechnology Inc; sc-271834, lot C3115), Phospho-YAP (Ser127) (Cell Signaling Technology; lot 13008), YAP (ABclonal; lot A1002), Cool/βPix(ARHGEF7) (Cell Signaling Technology; lot 4515), LATS1 (C66B5) (Cell Signaling Technology; lot 3477), β-Actin (Transgen Biotech; HC201-01), Myc tag(Bimake; A5968) were used for immunoblotting, Immunofluorescence and Immunohistochemistry. Anti-Myc magnetic beads were used for IP and was purchased from Biomake. Verteporfin was purchased from MedChemExpress (MCE).

shRNA, cDNA constructs and virus infection

shRNA constructs targeting human SHANK2 were designed and cloned into the pLKO.1 shRNA vector. shRNA design and cloning were performed according to protocols at http://portals.broadinstitute.org/gpp/public/. The hairpin targeting sequences were SHANK2#1: GCCAATCTCAAATAAGCCTTT, SHANK2#2: CGACCTCAACAAACCTCTTTA. Lentivirus was produced in 293T co-transfected with shRNA- pLKO.1, Δ8.9 and VSVG plasmids. Human SHANK2 cDNA was synthesized at GenScript and was cloned into retroviral pMSCV-IRES-GFP vector. Deletion of PDZ domain (624-717aa) from SHANK2 was achieved by overlap extension PCR. Retrovirus was produced in 293T co-transfected with retroviral vector and helper plasmids. All viruses were harvested at 48 h post-transfection. cDNAs of SHANK2, SHANK2 ΔPDZ and ARHGEF7 were also cloned into a transient transfection vector pcDNA 3.1 for immunoprecipitation experiment.

Immunofluorescence

Cells were seeded onto coverslips at low or high density. Cells were washed one time with PBS, fixed with 4% PFA for 15min and then washed three times with PBS, permeabilized with 0.25% TirtonX-100 for 15min. After permeabilization, cells were washed three times with PBS and blocked with 3% BSA for 2 hours. Then cells were incubated with YAP antibody (1:500 dilution, ABclonal) overnight at 4℃. The cells were then washed three times with PBS and incubated with secondary antibody (1:1000 dilution, Molecular Probes) for 1 hour. After washing three times with PBS, DAPI diluted with PBS were used to label the nucleus. Images were collected with Leica TCS SP8 equipped with LAS software.

RNA extraction and qPCR

Total RNA was extracted from cells or tumors with Trizol reagent (Ambion by life technology). RNA was transcribed to cDNA with the GoScript^{TM} Reverse

Transcription System (Promega). qPCR was performed with Go Taq^{R} qPCR Master Mix (Promega). The primers used for qPCR were as follows:

SHANK2-F: CTTTGGATTCGTGCTTCGAGG,

SHANK2-R: GACTCCAGGTACTGTAGGGC;

Human CTGF -F: CAGCATGGACGTTCGTCTG,

Human CTGF -R: AACCACGGTTTGGTCCTTGG;

Human CYR61-F: CTCGCCTTAGTCGTCACCC,

Human CYR61-R: CGCCGAAGTTGCATTCCAG;

Mouse CTGF-F: GGGCCTCTTCTGCGATTTC,

Mouse CTGF-R: ATCCAGGCAAGTGCATTGGTA;

Mouse CYR61-F: CTGCGCTAAACAACTCAACGA,

Mouse CYR61-R: GCAGATCCCTTTCAGAGCGG;

Human GAPDH-F: GGAGCGAGATCCCTCCAAAAT，

Human GAPDH-R: GGCTGTTGTCATACTTCTCATGG；

Mouse GAPDH-F: AGGTCGGTGTGAACGGATTTG，

Mouse GAPDH-R: GGGGTCGTTGATGGCAACA.

Immunoprecipitation

Anti-Myc magnetic beads were washed three times with washing buffer and suspended with PBS. Cells were lysed with lysis buffer for 10min at 4℃ and the lysate was incubated with beads overnight at 4℃. After that, beads were washed three times with washing buffer and boiled with same volume of 2×SDS loading buffer at 100℃ for 15mins, and supernatants were analyzed by Western Blotting using relevant antibodies.

Cell proliferation assay

0.05 million of cells were seeded at 6cm dish. At time of analysis, cells were digested with 0.5 ml trypsin enzyme, neutralized with 2ml complete media and cell number were analyzed by flow cytometry.

Colony formation assay

500 of 293T cells expressing vector control or exogenous SHANK2 were seeded in tissue culture plate and treated with DMSO or 2.5μM verteporfin for 24h after 6 days. After removal of verteporfin, cells were kept in culture for another two days and the resulting colonies were stained with 1 ml of 0.02% Crystal Violet.

Soft agar cell transformation assay

The protocol of Soft-Agar growth was kindly provided by professor Hongbin Ji. The bottom layer of 1% Agarose containing 0.67ml 3% Agarose and 1.34ml complete media was laid per well on 6-well plates. Then same volume of top layer was added after bottom layer formed. The top layer containing 0.4% Agarose is consisted of 0.27ml 3% Agarose, 0.63ml complete media, 0.1ml FBS and 1ml complete media, and 500 cells were seeded in the top layer. Every 3 days, 3-4 drops of complete media were added to each well. When colonies grew up, plates were stained with 0.5 ml of 0.005% Crystal Violet.

Xenograft

2 million of cells were suspended with cold PBS and injected into the flanks of BALB/c nude mice. Tumor sizes were monitored by caliper and calculated as: 1/2 ×length ×width ×width.

Hydrodynamic injection for liver cancer

We used Sleeping Beauty transposon to initiate liver tumorigenesis. The vector system was kindly provided by Prof. Xin Chen at UCSF.10μg p53R246S, 2.5μg Myc and 10μg vector or cDNA of SHANK2, or SHANK2 ΔPDZ plasmids, combined with 1μg Sleeping Beauty transposase plasmid were diluted into 2ml 0.9%NaCl and were injected to 7-week-old FVB/n mice by rapid tail vein injection within 6-8 seconds.

Immunohistochemistry assay

The liver tissues were fixed with 4% paraformaldehyde (PFA) 48h at 4℃ and were dehydrated and embedded for cryostat sectioning. Then the cryostat sections were incubated with YAP antibody (1:50 dilution, ABclonal) overnight at 4℃, and incubated with secondary antibody (1:100 dilution, Molecular Probes) for 1 hours. After washing three times with PBS, DAPI diluted with PBS were used to stain the nucleus. Images were collected with Leica TCS SP8 equipped with LAS software.

Statistics analysis

All statistical data were carried out with GraphPad Prism 6. The quantification data were statistically presented as average with the standard error of mean (SEM) and p-values of significance is calculated by Student’s T-test; * $P<0.05$, ** $P<0.01$, *** $P<0.001$, **** $P<0.0001$ and NS: not significant with $P>0.05$.

Supplementary data

Supplementary Table 1. ***Prosap*****'s homolog SHANK2 is focally amplified in human cancer.**

Analysis was done using the TCGA copy number portal. SHANK2 is highly amplified in human cancer. The amplification peak locus only contains SHANK2, suggesting its amplification is highly selected for during cancer formation.

	**% of focal amplification In epithelial cancers**	**# of genes in amplification peak**
SHANK2	11%	1 (SHANK2)
SHANK1	2%	6
SHANK3	2%	90

Supplementary Table 2. List of the top 100 most frequently amplified genes in human cancer. Data were acquired from COSMIC website.

Gene name	Gene Location	Number of cancer samples carrying gene amplification in COSMIC database
MYC	8:127738263..127740958	968
POU5F1B	8:127415867..127416946	929
TMEM75	8:127947618..127948034	896
FAM84B	8:126556457..126557389	847
NSMCE2	8:125102331..125366885	805
KIAA0196	8:125024617..125083898	797
MTSS1	8:124552992..124727955	796
COL14A1	8:120147843..120371231	795
SQLE	8:124999404..125021945	793
DEPTOR	8:119873847..120049704	790
ZNF572	8:124975641..124977858	789
FER1L6	8:123955999..124119790	786
TATDN1	8:124488594..124539046	785
NDUFB9	8:124539187..124549892	783
ANXA13	8:123681240..123737334	783
TMEM65	8:124313960..124372157	781
RNF139	8:124475110..124487644	780
FAM91A1	8:123768703..123812704	780
ADCY8	8:130780390..131040333	779
TRMT12	8:120538877..120811843	778
SNTB1	8:124450928..124452274	778
MRPL13	8:120396104..120445094	776
MTBP	8:120445471..120523336	774
KLHL38	8:123645739..123652926	774
HAS2	8:121614109..121641390	768
FBXO32	8:123503373..123541014	765
ASAP1	8:130054731..130401943	763
WDYHV1	8:123416850..123441415	762
ATAD2	8:123321134..123396357	761
TAF2	8:119731924..119832564	760
DSCC1	8:119834893..119855795	760
ZHX1	8:123253325..123255946	755
SAMD12	8:118379417..118621816	755
ENPP2	8:119557521..119638780	754
FAM83A	8:123182857..123207688	753
EXT1	8:117799712..118111046	753
COLEC10	8:119067282..119106191	753

C8orf76	8:123220103..123241346	752
TNFRSF11B	8:118924374..118951821	750
KCNQ3	8:129748501..129777587	750
GSDMC	8:132129262..132480532	750
WDR67	8:123072770..123151939	749
NOV	8:119416533..119423132	746
FAM49B	8:129842142..129879461	743
DERL1	8:123015447..123042122	742
TG	8:132867001..133134794	741
ZHX2	8:122951511..122954024	739
EFR3A	8:131904313..132010895	737
OC90	8:132024481..132049869	734
CSMD3	8:112224771..113436854	734
HHLA1	8:132063995..132105265	730
PHF20L1	8:132777829..132811134	729
MED30	8:117520877..117539978	729
WISP1	8:133191145..133227710	727
TMEM71	8:132710967..132758879	727
SLC30A8	8:117135328..117172681	725
LRRC6	8:132572306..132675493	725
SLA	8:133038524..133060160	721
EIF3H	8:116645006..116755797	721
NDRG1	8:133238878..133297237	720
C8orf85	8:116938244..116942701	718
TRPS1	8:115414023..115623637	717
ST3GAL1	8:133459764..133476024	717
RAD21	8:116847500..116866729	716
KHDRBS3	8:135457867..135647084	716
UTP23	8:116766604..116771842	715
ZFAT	8:134600334..134657720	696
ZFPM2	8:105318942..105803538	693
LOC51059	8:138132593..138367983	679
FAM135B	8:138132593..138367983	679
ANGPT1	8:107251855..107497558	679
RIMS2	8:103819508..104251820	678
OXR1	8:106679254..106750941	671
ABRA	8:106761037..106770190	669
TTC35	8:108443659..108486598	665
COL22A1	8:138589253..138883172	664
PKHD1L1	8:109362581..109530090	662
DPYS	8:104381198..104466920	661
LRP12	8:104490673..104588897	660
NUDCD1	8:109243009..109334010	657

TM7SF4	8:104348553..104356198	656
KCNV1	8:109968088..109974388	656
TRHR	8:109087513..109119455	655
RSPO2	8:107901075..108083648	655
SNX31	8:100573865..100649514	654
TMEM74	8:108784181..108785098	653
GRHL2	8:101492770..101666703	653
ENY2	8:109334469..109343481	653
SHANK2	11:70472869..70661767	652
LOC157567	8:100522555..100529833	652
ANKRD46	8:100522555..100529833	652
SYBU	8:109574906..109642947	651
EIF3E	8:108201885..108248702	651
EBAG9	8:109550825..109564559	650
PABPC1	8:100704298..100721583	646
YWHAZ	8:100920693..100948889	643
ZNF706	8:101200002..101201741	640
LOC51123	8:101200002..101201741	640
SPAG1	8:100162281..100241022	638
RNF19A	8:100258556..100288174	638

Supplementary Table 3. List of cancer samples carrying 11q13 amplification according to SHANK2 and Cyclin D1 status. Data were acquired from COSMIC website.

**Cancer samples that only amplify SHANK2**			
A253	TCGA-77-8148-01	TCGA-BR-4272-01	TCGA-G3-A3CJ-01
A431	TCGA-85-7699-01	TCGA-BR-4273-01	TCGA-G9-7509-01
HCE-4	TCGA-85-8048-01	TCGA-BR-6709-01	TCGA-G9-A9S0-01
HDQ-P1	TCGA-85-8072-01	TCGA-BR-8365-01	TCGA-GS-A9TZ-01
JHH-7	TCGA-85-8276-01	TCGA-BR-8367-01	TCGA-HB-A2OT-01
KYSE-140	TCGA-85-8287-01	TCGA-BT-A3PH-01	TCGA-HC-7212-01
KYSE-30	TCGA-85-A4QQ-01	TCGA-BT-A42C-01	TCGA-HC-7213-01
KYSE-410	TCGA-90-7766-01	TCGA-C5-A1BE-01	TCGA-HC-7818-01
OE21	TCGA-90-A4EE-01	TCGA-C8-A1HG-01	TCGA-HC-8266-01
PCI-15A	TCGA-92-7341-01	TCGA-C8-A1HK-01	TCGA-HC-A8D1-01
PCI-38	TCGA-93-7348-01	TCGA-CC-A8HT-01	TCGA-HD-7229-01
SCC-25	TCGA-94-A4VJ-01	TCGA-CD-8536-01	TCGA-IB-7890-01
TCGA-04-1346-01	TCGA-96-8169-01	TCGA-CJ-4635-01	TCGA-IG-A3Y9-01
TCGA-04-1347-01	TCGA-97-7553-01	TCGA-CN-4739-01	TCGA-IG-A4P3-01
TCGA-04-1542-01	TCGA-98-A538-01	TCGA-CN-4742-01	TCGA-IG-A51D-01
TCGA-05-4389-01	TCGA-A1-A0SE-01	TCGA-CN-5361-01	TCGA-IN-A7NT-01
TCGA-05-4398-01	TCGA-A2-A0CL-01	TCGA-CN-5363-01	TCGA-JW-A5VK-01
TCGA-05-4425-01	TCGA-A2-A0EQ-01	TCGA-CN-5365-01	TCGA-K4-A3WV-01
TCGA-05-4430-01	TCGA-A2-A0YK-01	TCGA-CN-5366-01	TCGA-KB-A93H-01
TCGA-06-1802-01	TCGA-A3-3311-01	TCGA-CN-5367-01	TCGA-KK-A8I5-01
TCGA-09-1668-01	TCGA-A3-3313-01	TCGA-CN-6013-01	TCGA-KK-A8II-01
TCGA-09-2050-01	TCGA-A3-3326-01	TCGA-CN-6020-01	TCGA-KK-A8IK-01
TCGA-13-1477-01	TCGA-A3-3336-01	TCGA-CN-6989-01	TCGA-KL-8344-01
TCGA-13-1504-01	TCGA-A3-3363-01	TCGA-CN-6994-01	TCGA-KU-A66T-01
TCGA-13-2065-01	TCGA-A5-AB3J-01	TCGA-CN-6995-01	TCGA-L5-A4OU-01
TCGA-13-2071-01	TCGA-A6-2675-01	TCGA-CN-6997-01	TCGA-L5-A8NI-01
TCGA-13-A5FU-01	TCGA-A7-A0DC-01	TCGA-CN-A498-01	TCGA-L5-A8NK-01
TCGA-14-0736-01	TCGA-A7-A26G-01	TCGA-CN-A49B-01	TCGA-L5-A8NQ-01
TCGA-14-0813-01	TCGA-A7-A26J-01	TCGA-CN-A49C-01	TCGA-L5-A8NS-01
TCGA-18-3411-01	TCGA-A7-A3IZ-01	TCGA-CN-A6V7-01	TCGA-L9-A5IP-01
TCGA-19-1388-01	TCGA-A7-A4SB-01	TCGA-CQ-5323-01	TCGA-L9-A8F4-01
TCGA-19-1788-01	TCGA-A8-A079-01	TCGA-CQ-6221-01	TCGA-LL-A441-01
TCGA-19-2620-01	TCGA-A8-A07L-01	TCGA-CQ-7067-01	TCGA-LN-A49V-01
TCGA-19-5952-01	TCGA-A8-A07W-01	TCGA-CQ-A4C6-01	TCGA-LN-A4A2-01
TCGA-20-1685-01	TCGA-A8-A082-01	TCGA-CQ-A4CA-01	TCGA-MN-A4N1-01
TCGA-22-5471-01	TCGA-A8-A09E-01	TCGA-CR-5247-01	TCGA-MT-A51W-01
TCGA-23-1107-01	TCGA-AA-3525-01	TCGA-CR-5248-01	TCGA-MT-A51X-01
TCGA-23-1120-01	TCGA-AA-3532-01	TCGA-CR-6474-01	TCGA-MU-A8JM-01

TCGA-23-1121-01	TCGA-AA-3548-01	TCGA-CR-6491-01	TCGA-MZ-A7D7-01
TCGA-23-2645-01	TCGA-AA-3552-01	TCGA-CR-7369-01	TCGA-NK-A5D1-01
TCGA-24-1104-01	TCGA-AA-3560-01	TCGA-CR-7371-01	TCGA-OL-A66O-01
TCGA-24-1471-01	TCGA-AA-3561-01	TCGA-CR-7383-01	TCGA-OL-A6VQ-01
TCGA-24-1563-01	TCGA-AA-3660-01	TCGA-CU-A3KJ-01	TCGA-OR-A5L2-01
TCGA-24-1604-01	TCGA-AA-3688-01	TCGA-CV-5430-01	TCGA-QK-A6IJ-01
TCGA-24-1923-01	TCGA-AA-3812-01	TCGA-CV-5973-01	TCGA-QK-A8ZA-01
TCGA-24-1927-01	TCGA-AA-3815-01	TCGA-CV-5979-01	TCGA-QK-AA3J-01
TCGA-24-2030-01	TCGA-AA-3821-01	TCGA-CV-6945-01	TCGA-R6-A6DN-01
TCGA-24-2295-01	TCGA-AA-3846-01	TCGA-CV-7095-01	TCGA-RD-A8N6-01
TCGA-24-2298-01	TCGA-AA-3947-01	TCGA-CV-7097-01	TCGA-RD-A8N9-01
TCGA-25-1877-01	TCGA-AA-3952-01	TCGA-CV-7178-01	TCGA-RY-A840-01
TCGA-25-2391-01	TCGA-AA-3955-01	TCGA-CV-7414-01	TCGA-T3-A92M-01
TCGA-25-2397-01	TCGA-AA-3956-01	TCGA-CV-7422-01	TCGA-TK-A8OK-01
TCGA-25-2409-01	TCGA-AA-3980-01	TCGA-CV-A45U-01	TCGA-TQ-A8XE-01
TCGA-26-1443-01	TCGA-AA-3986-01	TCGA-CX-7086-01	TCGA-UD-AABZ-01
TCGA-27-1835-01	TCGA-AA-3989-01	TCGA-D1-A169-01	TCGA-UD-AAC1-01
TCGA-28-1752-01	TCGA-AB-2812-03	TCGA-D3-A3CE-06	TCGA-UF-A7JA-01
TCGA-29-1783-01	TCGA-AB-2999-03	TCGA-D8-A1JC-01	TCGA-UF-A7JC-01
TCGA-2J-AAB4-01	TCGA-AC-A23H-01	TCGA-D8-A1X5-01	TCGA-UY-A8OD-01
TCGA-2J-AABT-01	TCGA-AC-A2BM-01	TCGA-D8-A1XF-01	TCGA-UY-A9PH-01
TCGA-2J-AABU-01	TCGA-AC-A2FB-01	TCGA-D9-A3Z3-06	TCGA-V1-A9ZG-01
TCGA-2L-AAQE-01	TCGA-AG-3583-01	TCGA-DA-A95Y-06	TCGA-V4-A9E5-01
TCGA-2Y-A9GX-01	TCGA-AJ-A2QK-01	TCGA-DD-AACA-01	TCGA-V5-A7RC-01
TCGA-2Y-A9HB-01	TCGA-AJ-A3EL-01	TCGA-DD-AACJ-01	TCGA-V5-AASW-01
TCGA-32-1977-01	TCGA-AN-A0AJ-01	TCGA-DD-AACO-01	TCGA-VG-A8LO-01
TCGA-35-3615-01	TCGA-AN-A0FW-01	TCGA-DD-AAD1-01	TCGA-VM-A8CA-01
TCGA-36-1577-01	TCGA-AN-A0XW-01	TCGA-DD-AADK-01	TCGA-VQ-AA6B-01
TCGA-36-1578-01	TCGA-AO-A03O-01	TCGA-DD-AADN-01	TCGA-VQ-AA6J-01
TCGA-36-2539-01	TCGA-AR-A0TZ-01	TCGA-DD-AADP-01	TCGA-VQ-AA6K-01
TCGA-36-2543-01	TCGA-AR-A1AK-01	TCGA-DD-AADS-01	TCGA-VR-A8EO-01
TCGA-39-5037-01	TCGA-AR-A2LJ-01	TCGA-DD-AAEG-01	TCGA-VR-A8ER-01
TCGA-3A-A9IZ-01	TCGA-B0-4828-01	TCGA-DD-AAEH-01	TCGA-VR-A8EU-01
TCGA-3H-AB3T-01	TCGA-B0-4836-01	TCGA-DD-AAVV-01	TCGA-VR-A8EW-01
TCGA-3H-AB3U-01	TCGA-B5-A11Q-01	TCGA-DK-A1A5-01	TCGA-VR-A8EX-01
TCGA-3M-AB47-01	TCGA-B6-A0I9-01	TCGA-DK-A3IM-01	TCGA-VR-A8EZ-01
TCGA-3U-A98F-01	TCGA-B6-A0IG-01	TCGA-DK-AA74-01	TCGA-VS-A8QM-01
TCGA-42-2582-01	TCGA-B9-4116-01	TCGA-DK-AA77-01	TCGA-W5-AA2G-01
TCGA-43-6771-01	TCGA-BA-4074-01	TCGA-DQ-7588-01	TCGA-W8-A86G-01
TCGA-46-3765-01	TCGA-BA-4075-01	TCGA-DT-5265-01	TCGA-WE-A8ZN-06
TCGA-46-3769-01	TCGA-BA-4078-01	TCGA-DX-AB2E-01	TCGA-WS-AB45-01
TCGA-4W-AA9T-01	TCGA-BA-5149-01	TCGA-DX-AB37-01	TCGA-WY-A858-01
TCGA-4Z-AA7S-01	TCGA-BA-6868-01	TCGA-E2-A109-01	TCGA-X6-A8C4-01

TCGA-4Z-AA86-01	TCGA-BA-6872-01	TCGA-E2-A15K-01	TCGA-XF-A9T4-01
TCGA-55-8203-01	TCGA-BB-4227-01	TCGA-E2-A15M-01	TCGA-XF-AAML-01
TCGA-56-8083-01	TCGA-BB-7863-01	TCGA-E2-A15R-01	TCGA-XN-A8T3-01
TCGA-57-1582-01	TCGA-BB-7871-01	TCGA-E2-A1IU-01	TCGA-XP-A8T6-01
TCGA-5T-A9QA-01	TCGA-BB-A5HU-01	TCGA-E9-A1N9-01	TCGA-XP-A8T8-01
TCGA-60-2715-01	TCGA-BH-A0BD-01	TCGA-E9-A1QZ-01	TCGA-XS-A8TJ-01
TCGA-60-2725-01	TCGA-BH-A0BV-01	TCGA-E9-A228-01	TCGA-XX-A899-01
TCGA-63-A5MJ-01	TCGA-BH-A0C3-01	TCGA-EA-A1QT-01	TCGA-YG-AA3P-06
TCGA-63-A5MT-01	TCGA-BH-A0DD-01	TCGA-EB-A44Q-06	TCGA-YL-A9WY-01
TCGA-63-A5MU-01	TCGA-BH-A0DI-01	TCGA-EB-A5SH-06	TCGA-YT-A95F-01
TCGA-66-2737-01	TCGA-BH-A0DZ-01	TCGA-EE-A2GD-06	TCGA-YY-A8LH-01
TCGA-66-2744-01	TCGA-BH-A0EI-01	TCGA-EJ-7792-01	TCGA-Z2-AA3V-06
TCGA-66-2767-01	TCGA-BH-AB28-01	TCGA-EJ-A8FU-01	TCGA-ZF-AA52-01
TCGA-66-2769-01	TCGA-BP-4337-01	TCGA-EK-A2IP-01	TCGA-ZF-AA53-01
TCGA-66-2778-01	TCGA-BP-4343-01	TCGA-EL-A3T2-01	TCGA-ZG-A9KY-01
TCGA-66-2782-01	TCGA-BP-4355-01	TCGA-EL-A3T9-01	TCGA-ZG-A9LY-01
TCGA-66-2789-01	TCGA-BP-4771-01	TCGA-EY-A72D-01	TCGA-ZP-A9CZ-01
TCGA-66-2790-01	TCGA-BP-4777-01	TCGA-FB-AAQ6-01	TE-10
TCGA-73-4676-01	TCGA-BP-4787-01	TCGA-FC-7708-01	TE-11
TCGA-75-5126-01	TCGA-BP-4983-01	TCGA-G2-A2ES-01	TE-6
TCGA-77-8131-01	TCGA-BP-4989-01	TCGA-G2-A3IB-01	TE-8
TCGA-77-8133-01	TCGA-BP-4994-01	TCGA-G2-AA3B-01	TE-9
**Cancer samples that only amplify CCND1**			
DMS-114	TCGA-A8-A06U-01	TCGA-DI-A2QU-01	TCGA-J9-A8CL-01
DSH1	TCGA-A8-A07B-01	TCGA-DX-A2J1-01	TCGA-L5-A88V-01
MFM-223	TCGA-AC-A3QQ-01	TCGA-E2-A14V-01	TCGA-LL-A7SZ-01
NCI-H1395	TCGA-AR-A24R-01	TCGA-E5-A4U1-01	TCGA-LN-A4A1-01
TCGA-2Y-A9H9-01	TCGA-BF-A9VF-01	TCGA-E9-A22D-01	TCGA-LN-A4A5-01
TCGA-44-6774-01	TCGA-BR-4259-01	TCGA-EB-A57M-01	TCGA-RD-A7C1-01
TCGA-49-6767-01	TCGA-CC-5264-01	TCGA-ER-A19W-06	TCGA-S5-AA26-01
TCGA-64-1678-01	TCGA-CC-A3M9-01	TCGA-EW-A1J3-01	TCGA-UF-A71E-01
TCGA-64-5815-01	TCGA-CD-5799-01	TCGA-EW-A2FW-01	TCGA-VQ-A8DU-01
TCGA-68-A59J-01	TCGA-CG-4455-01	TCGA-EW-A423-01	TCGA-XF-AAN8-01
TCGA-78-7542-01	TCGA-CV-6956-01	TCGA-G3-A25W-01	TCGA-Z6-A8JE-01
TCGA-98-8020-01	TCGA-DD-AADL-01	TCGA-HE-7130-01	
TCGA-A2-A0YF-01	TCGA-DD-AAE6-01	TCGA-HU-8610-01	
**Cancer samples that amplify both SHANK2 and CCND1**			
451Lu	TCGA-AC-A3OD-01	TCGA-CV-A460-01	TCGA-KQ-A41O-01
BFTC-905	TCGA-AC-A4ZE-01	TCGA-CV-A6JE-01	TCGA-L5-A88S-01
BHY	TCGA-AC-A6IX-01	TCGA-CV-A6JM-01	TCGA-L5-A8NF-01
BICR22	TCGA-AN-A03Y-01	TCGA-CV-A6JN-01	TCGA-L5-A8NW-01
CAL-39	TCGA-AN-A0FS-01	TCGA-CX-7082-01	TCGA-LD-A7W6-01

EPLC-272H	TCGA-AO-A0J3-01	TCGA-D3-A2J6-06	TCGA-LL-A7T0-01
ES-2	TCGA-AO-A0JD-01	TCGA-D3-A2JB-06	TCGA-LN-A49M-01
FADU	TCGA-AO-A0JG-01	TCGA-D3-A51E-06	TCGA-LN-A49R-01
HOS	TCGA-AO-A12F-01	TCGA-D3-A8GB-06	TCGA-LN-A49W-01
KU-19-19	TCGA-AR-A1AP-01	TCGA-D6-6825-01	TCGA-LN-A49Y-01
KYSE-180	TCGA-B6-A0IP-01	TCGA-D6-6826-01	TCGA-LN-A4A3-01
KYSE-510	TCGA-B6-A0WW-01	TCGA-D6-A4Z9-01	TCGA-LN-A4A4-01
MDA-MB-330	TCGA-BA-4077-01	TCGA-D6-A6ES-01	TCGA-LN-A4A6-01
MDA-MB-415	TCGA-BA-6871-01	TCGA-D7-A6EV-01	TCGA-LN-A4MR-01
NCI-H23	TCGA-BA-A4II-01	TCGA-D8-A1JI-01	TCGA-LN-A5U7-01
PCI-30	TCGA-BA-A6DD-01	TCGA-D8-A1Y2-01	TCGA-LN-A7HW-01
PE/CA-PJ15	TCGA-BG-A0M8-01	TCGA-DD-A4ND-01	TCGA-LN-A7HX-01
SCC-4	TCGA-BH-A0B1-01	TCGA-E2-A105-01	TCGA-LN-A7HZ-01
SW954	TCGA-BH-A0BR-01	TCGA-E2-A14O-01	TCGA-LN-A8I1-01
TCGA-10-0938-01	TCGA-BH-A0HL-01	TCGA-E2-A150-01	TCGA-MT-A67D-01
TCGA-13-0802-01	TCGA-BH-A0W3-01	TCGA-E2-A15A-01	TCGA-NC-A5HH-01
TCGA-13-1501-01	TCGA-BH-A18I-01	TCGA-E2-A2P6-01	TCGA-NC-A5HO-01
TCGA-18-3410-01	TCGA-BH-A18L-01	TCGA-E7-A5KF-01	TCGA-O2-A52S-01
TCGA-18-3412-01	TCGA-BH-A18R-01	TCGA-E7-A6MD-01	TCGA-P3-A6SX-01
TCGA-22-1011-01	TCGA-BH-A18T-01	TCGA-E9-A1N5-01	TCGA-P3-A6T3-01
TCGA-23-1116-01	TCGA-BH-A1ES-01	TCGA-E9-A247-01	TCGA-P3-A6T4-01
TCGA-2J-AABF-01	TCGA-BR-8058-01	TCGA-EB-A41B-01	TCGA-P3-A6T7-01
TCGA-36-1571-01	TCGA-C8-A12M-01	TCGA-EB-A82B-01	TCGA-QK-A6IH-01
TCGA-3C-AAAU-01	TCGA-C8-A12O-01	TCGA-EK-A2PI-01	TCGA-QK-AA3K-01
TCGA-42-2587-01	TCGA-C8-A12Q-01	TCGA-ER-A19Q-06	TCGA-R3-A69X-01
TCGA-52-7812-01	TCGA-C8-A12W-01	TCGA-ER-A19T-01	TCGA-R6-A6XQ-01
TCGA-55-1595-01	TCGA-C8-A12Y-01	TCGA-ER-A3ES-06	TCGA-RC-A7SF-01
TCGA-58-8387-01	TCGA-C8-A26W-01	TCGA-EW-A2FS-01	TCGA-S3-A6ZG-01
TCGA-58-8390-01	TCGA-C8-A26Z-01	TCGA-F7-8298-01	TCGA-UF-A7JV-01
TCGA-60-2726-01	TCGA-C8-A3M8-01	TCGA-FD-A3B5-01	TCGA-VQ-A8PU-01
TCGA-61-1724-01	TCGA-CD-8533-01	TCGA-FD-A62S-01	TCGA-VQ-A92D-01
TCGA-63-A5M9-01	TCGA-CD-A48C-01	TCGA-FP-8099-01	TCGA-VR-AA4G-01
TCGA-63-A5MS-01	TCGA-CN-4728-01	TCGA-FV-A4ZQ-01	TCGA-VR-AA7B-01
TCGA-66-2768-01	TCGA-CN-5364-01	TCGA-GC-A6I1-01	TCGA-VR-AA7D-01
TCGA-70-6723-01	TCGA-CN-6011-01	TCGA-GD-A3OS-01	TCGA-VR-AA7I-01
TCGA-77-7465-01	TCGA-CN-6012-01	TCGA-GM-A2DL-01	TCGA-VS-A8Q9-01
TCGA-77-8009-01	TCGA-CQ-5326-01	TCGA-GM-A3NW-01	TCGA-VS-A9UY-01
TCGA-85-8354-01	TCGA-CQ-6228-01	TCGA-GN-A4U7-06	TCGA-W5-AA2X-01
TCGA-85-A511-01	TCGA-CR-6478-01	TCGA-GV-A3JV-01	TCGA-WD-A7RX-01
TCGA-A1-A0SM-01	TCGA-CR-7377-01	TCGA-H4-A2HQ-01	TCGA-XF-AAMQ-01
TCGA-A2-A0CK-01	TCGA-CR-7388-01	TCGA-H7-8502-01	TCGA-XP-A8T7-01
TCGA-A2-A0ER-01	TCGA-CR-7399-01	TCGA-HD-7753-01	TCGA-XV-A9W2-01
TCGA-A2-A0EW-01	TCGA-CV-5431-01	TCGA-HF-7134-01	TCGA-XV-A9W5-01

TCGA-A2-A0T3-01	TCGA-CV-5440-01	TCGA-HQ-A5ND-01	TCGA-XX-A89A-01
TCGA-A2-A0YT-01	TCGA-CV-5441-01	TCGA-HU-A4H6-01	TCGA-Z6-A9VB-01
TCGA-A2-A3KC-01	TCGA-CV-6935-01	TCGA-IG-A3YA-01	TCGA-Z7-A8R6-01
TCGA-A7-A425-01	TCGA-CV-6950-01	TCGA-IG-A3YC-01	TCGA-ZD-A8I3-01
TCGA-A7-A5ZV-01	TCGA-CV-7102-01	TCGA-IG-A5S3-01	TCGA-ZF-A9R7-01
TCGA-A8-A06O-01	TCGA-CV-7235-01	TCGA-IG-A6QS-01	TCGA-ZG-A9LS-01
TCGA-A8-A086-01	TCGA-CV-7247-01	TCGA-IG-A97I-01	TE-15
TCGA-A8-A08G-01	TCGA-CV-7423-01	TCGA-IQ-A61H-01	TK10
TCGA-A8-A09C-01	TCGA-CV-7432-01	TCGA-J2-A4AD-01	
TCGA-A8-A0A6-01	TCGA-CV-7435-01	TCGA-JY-A93E-01	
TCGA-A8-A0A9-01	TCGA-CV-A45X-01	TCGA-K1-A42X-01	

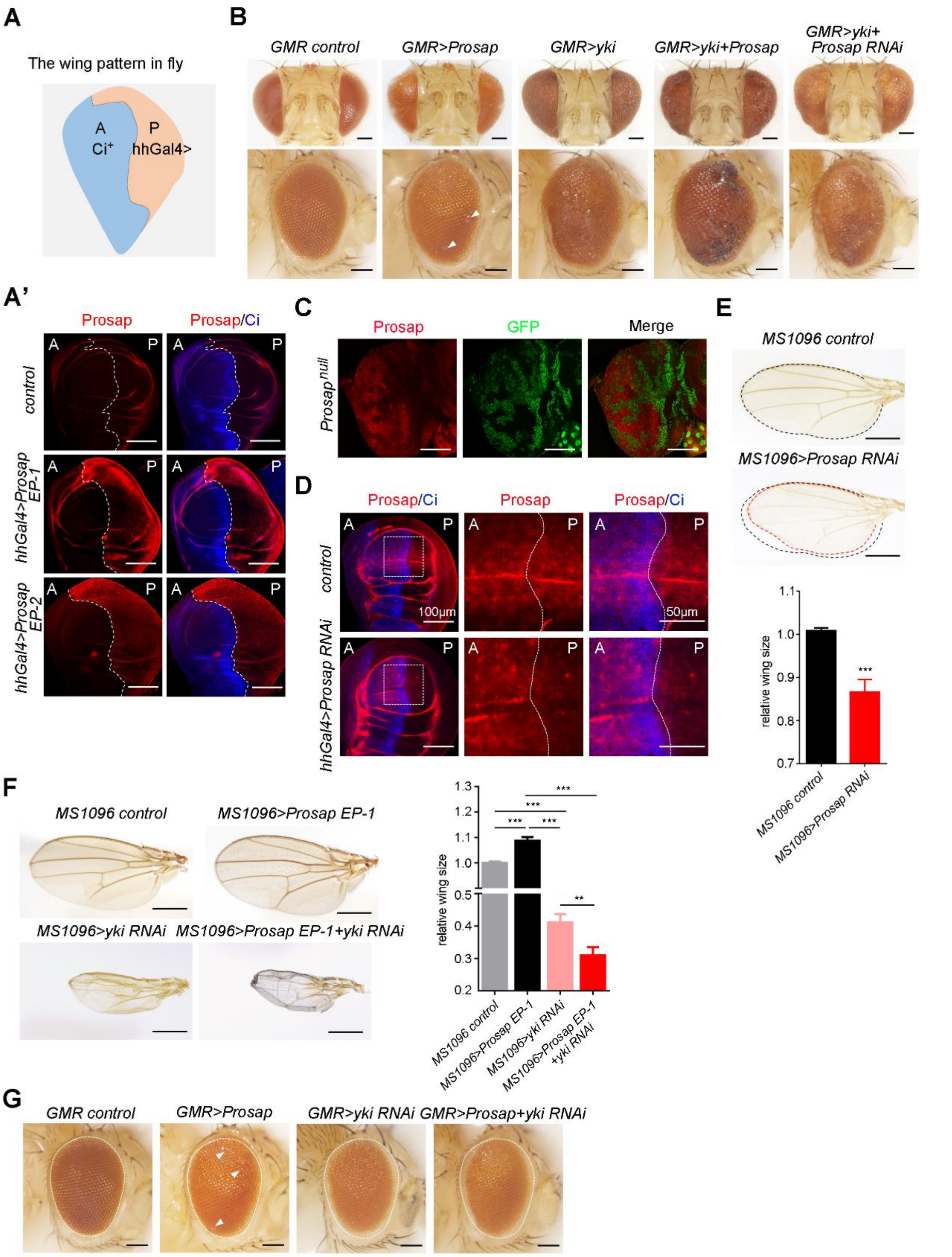

Supplementary Figure 1. ***Prosap*** **overexpression enhances tissue overgrowth via Hippo pathway in** ***Drosophila.***

A, A' In *Prosap EP-1* and *EP-2* lines, p element insertion enabled overexpression of *Prosap*. The upper panel (A) is a schematic representation of *Drosophila* third-instar larval wing discs showing *Ci* and *hhGal4* expression pattern. A,

anterior compartment (*Ci*$^{+}$); P, posterior compartment (*hhGal4*$^{+}$). The dashed line indicated the A/P boundary. The lower panels in (A') are representative images of *Drosophila* third-instar larval wing discs of indicated genotypes. Increased immunostaining of Prosap (red) was observed in the posterior wing discs of *hhGal4-Prosap EP-1* and *hhGal4 Prosap EP-2*. Scale bars: 100μm (A').

B *Prosap* overexpression significantly enhanced the eye overgrowth phenotypes induced by *yki* overexpression. Shown are side views and dorsal views of *Drosophila* eyes of indicated genotypes. Expression of *Prosap* and *yki* in *Drosophila* eyes was driven by *GMR-Gal4*. In the second column, arrowheads marked small bulging areas of cell overgrowth in *Prosap*-overexpression eye. Images in the last column shows that *Prosap* RNAi failed to suppress eye overgrowth phenotype induced by *yki* overexpression. Scale bars: 100μm.

C, D Immunostaining of endogenous *Prosap* expression in eyes and wings of control fly. In (C), we used the mosaic analysis with a repressible cell marker (MARCM) system (Lee and Luo, 2001) to generate GFP-positive, *Prosap*-null cells. In GFP-negative regions where endogenous *Prosap* gene remained intact, Prosap immunostaining signals were observed. Scale bars: 100μm. In (D), *Prosap* RNAi was expressed in the posterior wing discs, where a reduction of Prosap immunostaining signals was observed. This indicates that the endogenous *Prosap* gene is expressed in *Drosophila* wings. Scale bars: 100μm.

E Expression of *Prosap* RNAi reduced the wing size. Shown are representative images of control wings or wings expressing *Prosap* RNAi driven by *MS1096-Gal4*. Scale bars: 500μm. In the lower panel, data represent mean ± SEM from results of three independent experiments; n=15. P value was calculated by Students' t test; *** P<0.001.

F Prosap's pro-growth function depended on Yki. Shown are representative images of control wings or wings expressing *Prosap EP-1*, *yki* RNAi, *or yki* RNAi + *Prosap EP-1* driven by *MS1096-Gal4*. Scale bars: 500μm. In the right panel, data represent mean ± SEM from results of three independent experiments; n=9. P value was calculated by Students' t test; **P<0.01. ***P<0.001. The *yki* RNAi + *Prosap EP-1* wings are often folded, which may partially contribute to the observation that such wings appear smaller than *Yki* RNAi wings.

G *Yki* RNAi reduced eye overgrowth phenotype of *Prosap*-overexpressing *Drosophila*. Shown are representative images of eyes of indicated genotypes. In the second column, arrowheads marked small bulging areas of cell overgrowth in *Prosap*-overexpression eye. Such areas were reduced in *Prosap*-overexpressing, *yki* RNAi eyes, which were more smooth and smaller in size. Scale bars: 100μm.

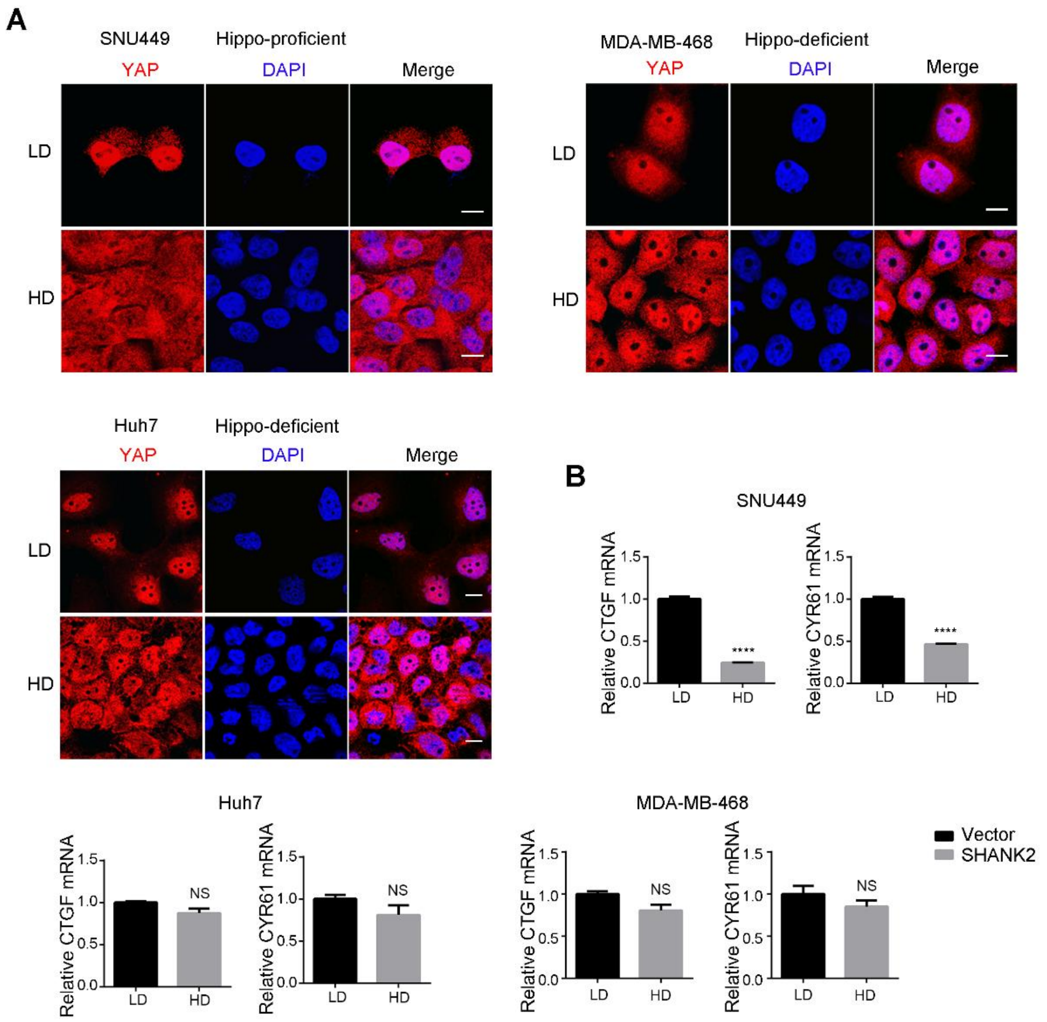

Supplementary Figure 2. Hippo signaling status in indicated human cell lines.

A Hippo pathway status were analyzed in SNU449 (Hippo-proficient), MDA-MB-468 and Huh7 (both Hippo-deficient) at low or high cell density (LD or HD). In Hippo-deficient cancer cell lines, YAP remained in nucleus at high cell density. Scale bars:10μm.

B Expression level of CTGF and CYR61 in SNU449 (Hippo-proficient), MDA-MB-468 and Huh7 (both Hippo-deficient) at low or high cell density (LD or HD). The expression level of CTGF and CYR61 was analyzed by qPCR. Data represent mean ±SEM from results of three independent experiments. P value was calculated by Students' t test; ****P<0.0001.

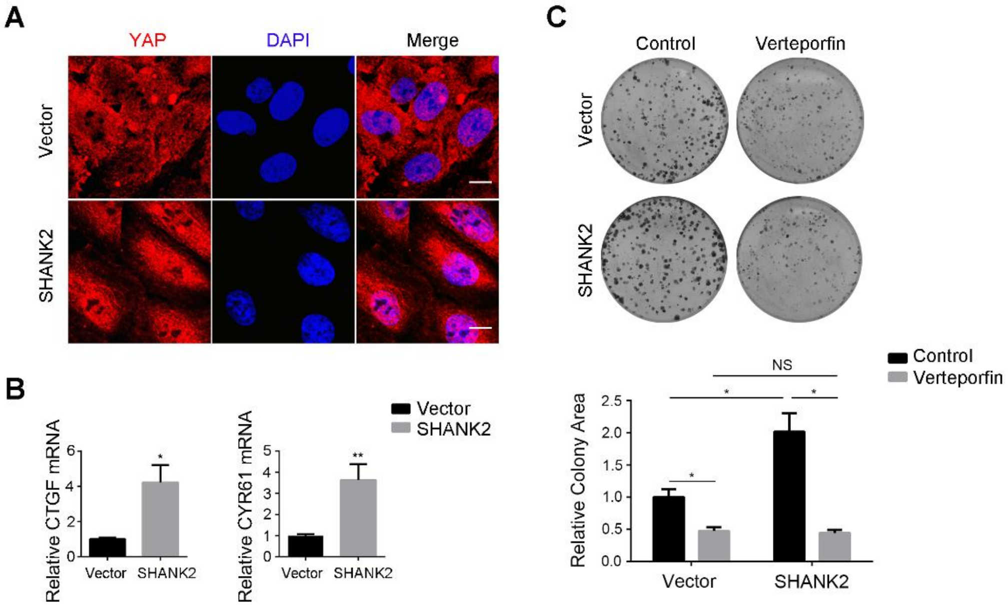

Supplementary Figure 3. SHANK2 overexpression causes deregulation of YAP.

A In Hippo-proficient cell line SNU449, YAP was localized at cytoplasm at high cell density, whereas ectopic expression of SHANK2 caused YAP to remain in nucleus at high cell density. Scale bars:10µm.

B SHANK2-overexpressing 293T cells exhibited increased CTGF and CYR61 expression when cells are cultured in high density. Data represent mean ± SEM from results of three independent experiments. P value was calculated by Students' t test; *P<0.05, **P<0.001.

C YAP1 inhibitor verteporfin suppressed SHANK2's ability to promote cell growth. 500 of 293T cells expressing vector control or exogenous SHANK2 were seeded in tissue culture plate and treated with DMSO or 2.5µM verteporfin for 24h. After removal of verteporfin, cells were kept in culture and the resulting colonies were stained with 1 ml of 0.02% Crystal Violet. In the bottom panel, data represent mean ± SEM from results of three independent experiments. P value was calculated by Students' t test; *P<0.05

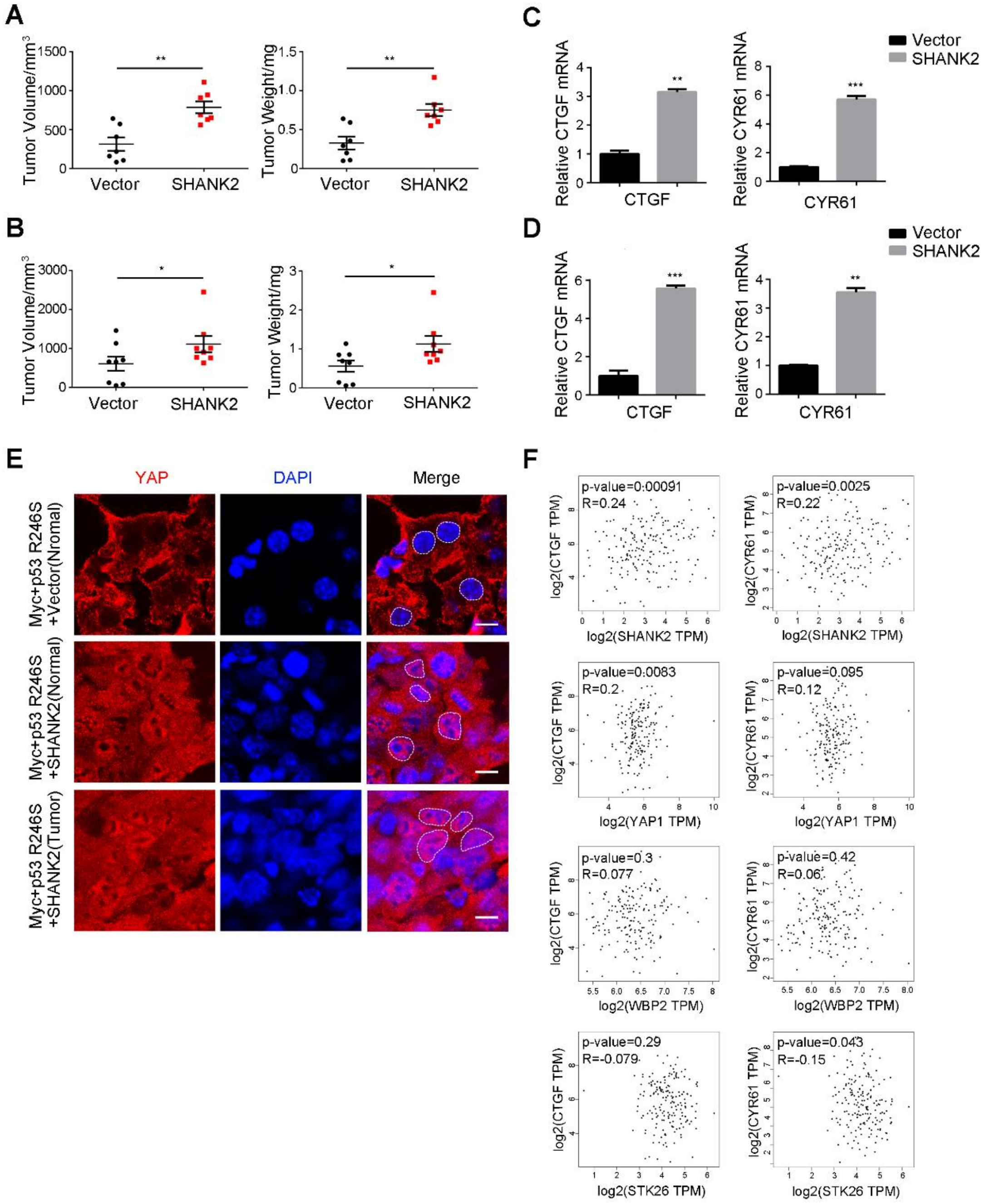

Supplementary Figure 4. SHANK2 promotes tumor growth.

A 2 million of 293T cells expressing vector control or exogenous SHANK2 were transplanted into nude mice. Tumor volume and tumor weight were measured at Day22 post injection. Data represent mean ± SEM; n=7. P value was calculated by Students' t test; ** $P<0.01$.

B 2 million of CommA-Dβ mouse mammary cells expressing vector control or exogenous SHANK2 were transplanted into the fat pad of breast of nude mice. Tumor volume and tumor weight were measured at Day41 post injection. Data

represent mean ± SEM; n=8. P value was calculated by Students' t test; *P<0.05.

C, D Expression of SHANK2 promoted YAP activity. The expression level of YAP transcriptional target genes CTGF and CYR61 in tumors arose form 293T(C) and CommA-Dβ(D) were analyzed by qPCR. Data represent mean ± SEM from results of results of three independent experiments. P value was calculated by Students' t test; **P<0.01, *** P<0.001.

E YAP was localized in nucleus in SHANK2-overexpressing normal liver cells and liver tumors. Vector control or exogenous SHANK2 combined with p53R246S and Myc were hydrodynamically injected into the tail vein of mice. Shown are immunostaining images of Yap (red) and DAPI (blue) of liver sections of the control group (normal liver) and SHANK2 overexpressing group (normal liver and liver tumor). In the right panel, several nuclei were marked by white dotted lines to show nuclear or cytoplasmic localization of YAP. Scale bars:10μm.

F Correlation of SHANK2 and CTGF, CYR61 expression in esophageal carcinoma (ESCA). The TCGA ESCA datasets were used for this analysis. Correlations between CTGF, CYR61 with YAP and two established positive regulators of YAP (WBP2 and STK26) were also show in comparison.

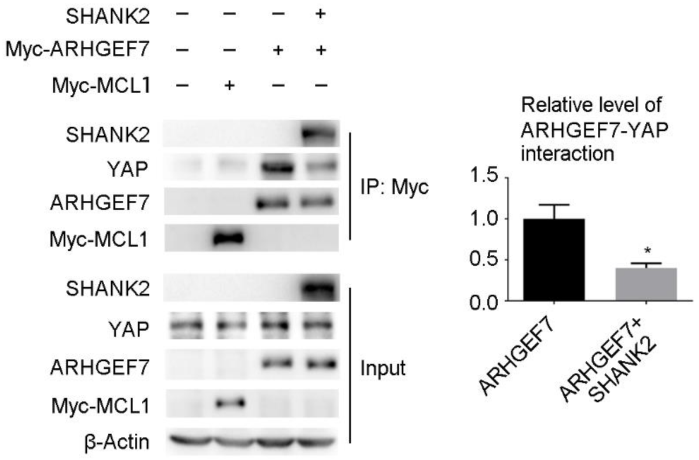

Supplementary Figure 5. SHANK2 overexpression reduced ARHGEF7-YAP interaction.

Co-immunoprecipitation experiments were done in 293T cells and Myc-MCL1 was used as negative control. The relative amount of ARHGEF7-YAP interactions were quantitated in the right panel as mean ±SEM from results of three independent experiments. P value was calculated by Students' t test; * $P<0.05$.

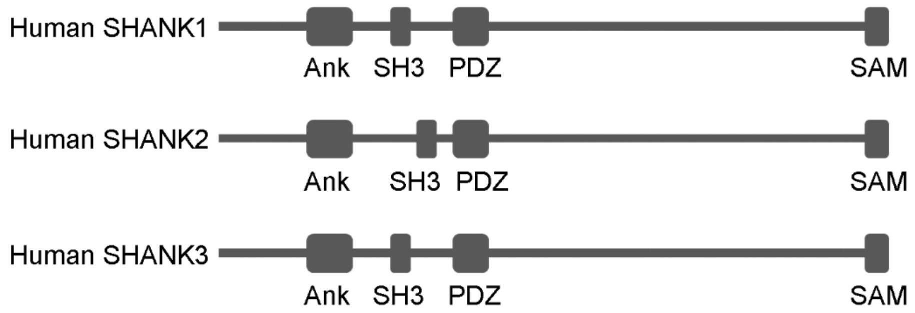

Supplementary Figure 6. Schematic representation of protein domains in human SHANK1, SHANK2 and SHANK3.
